# Z source based switched capacitor nine level boost inverter with a modified modulation strategy

**DOI:** 10.1038/s41598-024-79839-5

**Published:** 2024-11-22

**Authors:** Ahmed R. Hasouna, Sabry A. Mahmoud, Awad E. El-Sabbe, Dina S. M. Osheba

**Affiliations:** Electrical Engineering Department, Faculty of Engineering, Menoufia University, Shebin El-Kom, 32511 Egypt

**Keywords:** Multilevel inverter (MLI), ZS-MLI, Switched capacitor (SC), Modulation scheme, Capacitors soft charging, Engineering, Electrical and electronic engineering

## Abstract

This article presents a Z source (ZS) based switched capacitor multilevel inverter (SC-MLI) with low capacitors charging inrush currents utilizing a modified modulation strategy. The topology generates nine output voltage levels with quadruple voltage gain utilizing eight power switches, five capacitors, two inductors, four discrete diodes, and a single DC source. The voltage gain can be further increased by applying shoot through (ST) states to the frontend ZS-network. Owing to its high voltage gain, the topology is suitable for low voltage renewable energy sources such as photovoltaics (PV), and fuel cells (FC). A modified modulation strategy is presented based on the conventional level shifted sinusoidal pulse width modulation (LS-SPWM). The proposed modulation strategy improves the dc-link utilization at higher modulation index. A detailed mathematical analysis is provided for the gain calculation based on the modified modulation strategy. A comparative study is implemented to verify the merits of the proposed topology. A simulation model is implemented utilizing MATLAB / Simulink to verify the topology characteristics. The power losses and efficiency calculations are presented using Altair PSIM. Furthermore, an experimental prototype is constructed to verify the simulation findings. The topology is tested with different loading conditions at numerous modulation index values and ST duty ratios. Furthermore, step change conditions are implemented to test the topology response. The effect of the ZS network on the switched capacitors charging inrush currents is validated. The proposed topology shows an overall advantage in terms of the output characteristics as well as the hardware requirements.

## Introduction

The integration of renewable energy sources such as photovoltaic (PV), and fuel cells (FC), has been increasingly vital in the face of diminishing energy resources, and the global trend to mitigate environmental pollution. However, the low voltage issue of such sources hinders their wide range deployment. Conventionally, a power electronic converter is required to integrate low dc renewable energy sources into a higher voltage ac grid applications. Hence, a two-stage conversion process takes place to step up the input dc voltage before it is applied to a DC/AC inverter, imposing higher losses, lower efficiency, and more complicated conversion process. A well-known solution to implement a single stage conversion with voltage boosting capability is to utilize ZSI^1^. However, such topologies fall into the two-level inverters category, indicating higher output THD, and higher voltage stress on the power switches. Multilevel inverters (MLI) offer a wide range of features including lower THD, lower voltage stresses, lower $\:dv/dt$, and less common mode voltage (CMV)^2^. Consequently, numerous studies have investigated the incorporation of ZSI along with MLI topologies in terms of the power circuit, or the utilized modulation scheme^3^.

Cascaded H Bridge (CHB – MLI) has the advantage of modularity, and high reliability. However, it utilizes separate isolated DC sources which may be more suitable for renewable energy applications such as photovoltaic sources known for their low voltage issues. Consequently, the concept of Z – source (ZS), and quasi-Z source (QZS) networks incorporation with multilevel inverters has been introduced to address this issue through boosting the dc–link voltage $\:{V}_{dc-link}$ applied to the MLI. In^4^, a CHB-QZSI is proposed with a QZS network at the front end of each HB module. The topology can boost the input voltage by implementing a shoot through (ST) at the zero states. To control the duty ratio of the ST $\:\left(d\right)$, a modified modulation strategy is proposed which utilizes both phase shifted SPWM (PS-SPWM) modulation scheme as well as simple boost control (SBC). In^5^, a quasi-cascaded H-bridge five level inverter is proposed utilizing PS-SPWM. Compared to^4^, it utilizes fewer passive components as it utilizes two boost modules employing one capacitor and one inductor for each module. However, a voltage control technique is required to stabilize the utilized capacitors voltages, which further increases the control scheme complexity^6^. proposes another five-level cascaded inverter utilizing quasi switched boost inverters (QSBI). Compared to regular ZSI based topologies, it requires one less inductor and one less capacitor per boosting module. Consequently, it requires only 10 power switches, two capacitors, two inductors, four discrete diodes, and two isolated DC sources. In^7^, a modified quasi-Z-source (MQZS) network is utilized with CHB-MLI to reduce the number of required inductors and improve the quasi-network boost factor. To balance the two series capacitor voltages of the topology, and regulate the dc-link voltage, a modified phase shift modulation scheme is implemented based on alternative phase opposition disposition (APOD). However, the topology utilizes two QZS networks per module, which further increases the passive components requirements.

The aforementioned topologies implement relatively different modulation techniques. However, they share the common modulation principle that incorporates PS-SPWM scheme utilized for CHB-MLI with SBC of ZSI. Mainly a ST control signal $\:{V}_{SH}$ is compared with a high frequency (HF) triangular signals $\:{V}_{tria}$ with half the carrier amplitude, and twice its frequency. The switches control signal is generated by comparing sinusoidal control signals with the carrier signals. Then the ST states are inserted by inserting the ST control signals with the power switches control signals using OR gates^8^. The main issue with such approach is that the ST control signal is limited by the amplitude of the sinusoidal switches control signals (i.e., cannot be lower than $\:{V}_{control}$). Hence, the duty ratio becomes limited to the amplitude modulation index $\:{M}_{a}$ to be $\:(1-{M}_{a})$^9^. Consequently, it becomes essential to reduce $\:{M}_{a}$ for higher boosting factor in case of low voltage sources, which reduces the output power quality, and reduces the dc-link utilization of the converter. Numerous studies addressed this issue to improve the voltage gain versus the modulation index through modified configurations of passive elements at the expense of the higher hardware requirements^10–12^. Others have presented modified modulation strategies to improve the modulation index issue with ZSI/QZSI topologies^8,13–15^. However, they are still based on PS-SPWM which can be mainly implemented with CHB based MLI.

ZS networks integration to neutral point clamped MLI (NPC-MLI) have also been investigated in various studies in literature^16^. In^17^, a single stage ZS based three-level NPC-MLI is proposed. The topology requires two isolated DC sources as well as two ZS-networks, increasing the passive elements requirements as well as the converter cost and volume. A modified topology is also presented in^17^ to utilize single ZS-network. However, it still requires two isolated DC sources. Other topologies utilize high frequency coupling transformer at the front-end of the NPC-MLI in order to utilize only one DC source and improve the dc utilization^18^. Nevertheless, it comes at the cost of system size, as well as the relatively higher transformer losses.

Based on the previous discussion, it can be observed that multiple topologies have been developed incorporating ZSI with CHB-MLI and NPC-MLI. However, the number of output voltage levels is limited and requires multiple ZS-networks per circuit. Additionally, the voltage gain in such topologies is provided solely by the ZS-network that requires additional passive components to improve its boosting capability^19^.

When it comes to switched capacitors based MLI (SC-MLI), they provide multiple operation features compared to the classical MLI topologies. For example, SC-MLI offer lower switches count per output voltage levels^20^ and inherent boosting capability^21^. In^22^ a nine-level SC-MLI is presented with twofold voltage gain utilizing two dc sources with relatively high Total Standing Voltage (TSV). In^23^, a single source nine-level inverter is presented with quadruple voltage gain and low TSV. However, it requires higher switches count which increases its cost. To address the SC charging inrush currents^24^, presents a nine-level inverter with twofold voltage gain. However, the topology suffers from high switches voltage stress, and high hardware requirements leading to higher implementation cost^25^. presented a nine-level inverter with reduced switches count. However, it suffers from low voltage gain and unsuppressed capacitors inrush currents. In^26^, a low switches count nine-level inverter is presented with two dc sources. Nevertheless, it suffers from low voltage gain of unity and the switched capacitor inrush currents are not addressed.

Based on the aforementioned features of SC-MLI, incorporating ZSI with SC-MLI would present various benefits. For instance, SC-MLI topologies can be modified to operate utilizing only one DC source. Hence, only one ZS-network is required at the front-end of the MLI. Additionally, higher voltage gain is acquired due to the contribution of both ZS -network ST, as well as the SC aggregation. Finally, the SC inrush currents that are considered to be a major issue in SC-MLI, can be greatly suppressed through the ZS-network inductors.

The ZS incorporation with SC-MLI have not been well investigated in literature since^27^ presented a quasi ZS fed SC-MLI. In^27^ a single phase 7-level QZS-SC-MLI is presented utilizing 8 power switches, two discrete diodes, and two switched capacitors (SC). The classical front-end QZS-network requires two inductors, two capacitors, and a discrete diode. The modulation scheme utilized in^27^ is a combination of LS-SPWM and SBC that compares a positive and negative ST control signal with the upper and lower triangular carriers, respectively. Similar to modulation techniques utilized with ZSI, the ST control signal cannot be lower than the sinusoidal modulating signal, and the ST duty ratio is limited to the modulation index. For instance, at a unity modulation index, no ST can be applied, and hence no voltage boosting is acquired from the QZS network. Additionally, this conventional modulation strategy cannot be easily applied with higher voltage levels topologies that require more triangular carrier signals in their modulation scheme.

To address the aforementioned issues, this article presents a nine-level ZS network incorporated SC-MLI. The proposed topology is capable of integrating various ZS network variants (e.g., ZS, QZS, QSB, etc.), providing flexible connections and a broad range of output characteristics that reflect the diverse features of these networks. A modified modulation strategy is presented to insert ST states with higher duty ratio at high modulation index, offering better dc-link utilization. The topology is analyzed, simulated, and tested experimentally to verify the simulation results.

The rest of this article is organized as follows: Sect. 2 presents the topology construction and its modes of operations. Section 3 presents the proposed modulation strategy and its effect on the ZS boosting calculations. Section 4 presents the circuit components design and parameters calculations. Section 5 addresses the topology power loss analysis and its efficiency calculations. Section 6 presents a comparative study between the proposed topology and other similar topologies in literature. Section 7 presents the topology simulation, and experimental results. Finally, the conclusion is stated in Sect. 8.

**Fig. 1:**
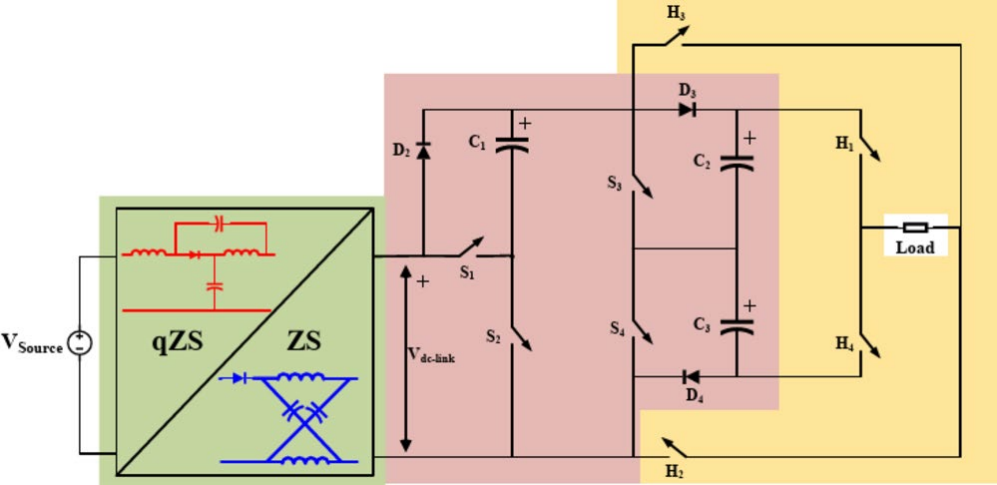
Proposed topology.

## Proposed topology and modes of operation

### Topology construction

The proposed topology shown in Fig. 1 utilizes a single DC source with a ZS/QZS-network consisting of two capacitors, two inductors, and one diode. The SC network utilizes 8 power switches, three switched capacitors, and three discrete diodes. The topology can be classified into three distinct parts as follows:

#### Impedance network

First, a basic conventional LC impedance network derived from the ZSI is utilized at the front-end of the converter. Different ZS/QZS network variants can be incorporated depending on the features required to be included in the topology. However, for general proposal, the conventional ZS- LC impedance network is adopted in the following analysis. As shown in Fig. 1, an LC network of two inductors, and two capacitors is utilized to provide a constant dc-link voltage $\:{V}_{dc-link}$ for the SC-MLI. This network can operate in either ST or NST modes. When both switches $\:{S}_{1}$, $\:{S}_{2}$ are turned ON at the same time in ST mode, a short circuit is applied at the impedance network causing both inductors $\:{L}_{m1}$, $\:{L}_{m2}$ to be charged, and $\:{V}_{dc-link}$ is zero. When only one of the two switches $\:{S}_{1}$, $\:{S}_{2}$ is turned ON in NST mode, the impedance network diode $\:{D}_{1}$ is forward biased. Consequently, the stored energy is transferred to the load. It is worth noting that during NST states, the switched capacitors SC are charged through $\:{L}_{m1}$, $\:{L}_{m2}$ which reduces the capacitors inrush charging currents.

#### Switched capacitor network

SC-Network consists of an array of capacitors and power switches. As shown in Fig. 1, it consists of two cascaded SC modules. The first module is a Series Parallel SC network (SPSC) which employs a capacitor $\:{C}_{1}$and two switches $\:{S}_{1}$, $\:{S}_{2}$. Switching ON $\:{S}_{2}$ charges $\:{C}_{1}$ with $\:{V}_{DC}$ through $\:{D}_{2}$. However, switching ON $\:{S}_{1}$ causes $\:{D}_{2}$ to be reverse biased. Hence, both the DC supply and $\:{C}_{1}$ are cascaded to create an output voltage of $\:2{V}_{dc-link}$, as it is the sum of $\:{V}_{dc-link}$ and $\:{V}_{C1}$. The second module is a SC voltage doubler network consisting of two capacitors ($\:{C}_{2}$, $\:{C}_{3}$), two switches ($\:{S}_{3}$, $\:{S}_{4}$), and two diodes ($\:{D}_{3}$, $\:{D}_{4}$). Each capacitor can be charged with $\:2{V}_{dc-link}$ as both the source and $\:{C}_{1}$ contributes to the charging process.

**Table 1:** Switching states during NST states.

$\:{V}_{out}$	$\:{S}_{1},\:\stackrel{-}{{S}_{2}}$	$\:{S}_{3},\:\stackrel{-}{{S}_{4}}$	$\:{H}_{1},\stackrel{-}{{H}_{4}}$	$\:{H}_{2},\stackrel{-}{{H}_{3}}$	$\:{C}_{1}$	$\:{C}_{2}$	$\:{C}_{3}$
-$\:4{V}_{dc}$	1	0	0	0	$\:\downarrow\:$	$\:\uparrow\:$	$\:\downarrow\:$
-$\:3{V}_{dc}$	0	0	0	0	$\:\uparrow\:$	-	$\:\downarrow\:$
-$\:2{V}_{dc}$	1	1	0	0	$\:\downarrow\:$	-	$\:\uparrow\:$
-$\:{V}_{dc}$	0	1	0	1	$\:\uparrow\:$	-	$\:\downarrow\:$
$\:{0}^{-}$	1	0	1	0	$\:\downarrow\:$	-	$\:\uparrow\:$
$\:{0}^{+}$	1	1	0	1	$\:\downarrow\:$	$\:\uparrow\:$	-
$\:{V}_{dc}$	0	0	1	0	$\:\uparrow\:$	$\:\downarrow\:$	-
$\:{2V}_{dc}$	1	0	1	1	$\:\downarrow\:$	$\:\uparrow\:$	-
$\:{3V}_{dc}$	0	1	1	1	$\:\uparrow\:$	$\:\downarrow\:$	-
$\:4{V}_{dc}$	1	1	1	1	$\:\downarrow\:$	$\:\downarrow\:$	$\:\uparrow\:$

($\:\uparrow\:$) denotes charging of the capacitor, ($\:\downarrow\:$) denotes discharging of the capacitor.

#### Polarity generation

Finally, an H-bridge is utilized to invert the output voltage polarity and hence produce an AC output voltage. Utilizing four switches ($\:{H}_{1},{H}_{2},\:{H}_{3},\:{H}_{4}\:$), operating in complementary manner as follows: $\:\left\{{H}_{1},\:\stackrel{-}{{H}_{4}}\right\},\:\{{H}_{2},\:\stackrel{-}{{H}_{3}}\}$. It is worth noting that only $\:\{{H}_{1},{H}_{4}\}$ have a maximum voltage stress (MVS) equal to the maximum output voltage $\:4{V}_{dc-link}$. While $\:\{{H}_{2},{H}_{3}\}$ have a MVS of $\:2{V}_{dc-link}$, resulting in lower TSV.

### Operation in NST states

During these states only one switch of $\:{S}_{1}$, $\:{S}_{2}$ are switched ON at a time to either charge or discharge the capacitors in the SC network. The NST switching states can be summarized in Table 1, while Fig. 2 illustrates their different modes of operation during the positive half cycle. It can be observed that the topology is capable of generating four different voltage levels in each half cycle ($\:\pm\:{V}_{dc},\pm\:{2V}_{dc},\pm\:{3V}_{dc},\pm\:{4V}_{dc}$), which produces nine output voltage levels, taking the zero-voltage level ($\:{0}^{\pm\:}$) into consideration. For simplicity the positive half cycle modes of operation illustrated in Fig. 2 can be explained as follows:

1. $\:{V}_{out}=+{V}_{dc}$: $\:{S}_{2}$ is switched ON to charge $\:{C}_{1}$ through $\:{D}_{2}$ from the supply to a voltage level of $\:{V}_{dc}$, while $\:{C}_{2}\:$discharges into the load through $\:{H}_{1},\:{H}_{3}$ so that the output voltage is $\:{{V}_{out}=V}_{c2}-{V}_{dc}={V}_{dc}$.
2. $\:{V}_{out}=+{2V}_{dc}$: $\:{S}_{1},{S}_{4}$ are switched ON to charge $\:{C}_{2}$ with $\:2{V}_{dc}$ as both the source and $\:{C}_{1}$ take part in the charging process $\:{V}_{c2}={V}_{dc}+{V}_{c1}$. While $\:{H}_{1},{H}_{2}$ are switched ON to supply the load.
3. $\:{V}_{out}=+{3V}_{dc}$: $\:{S}_{2}$ is switched ON to charge $\:{C}_{1}$ from the supply through $\:{D}_{2}$, while $\:{S}_{3}$ is switched ON to cascade $\:{C}_{2}$ with the source to supply the load with $\:3{V}_{dc}$ through $\:{H}_{1},\:{H}_{2}$, $\:{V}_{out}={V}_{dc}+{V}_{c2}$.
  **Fig. 2:**
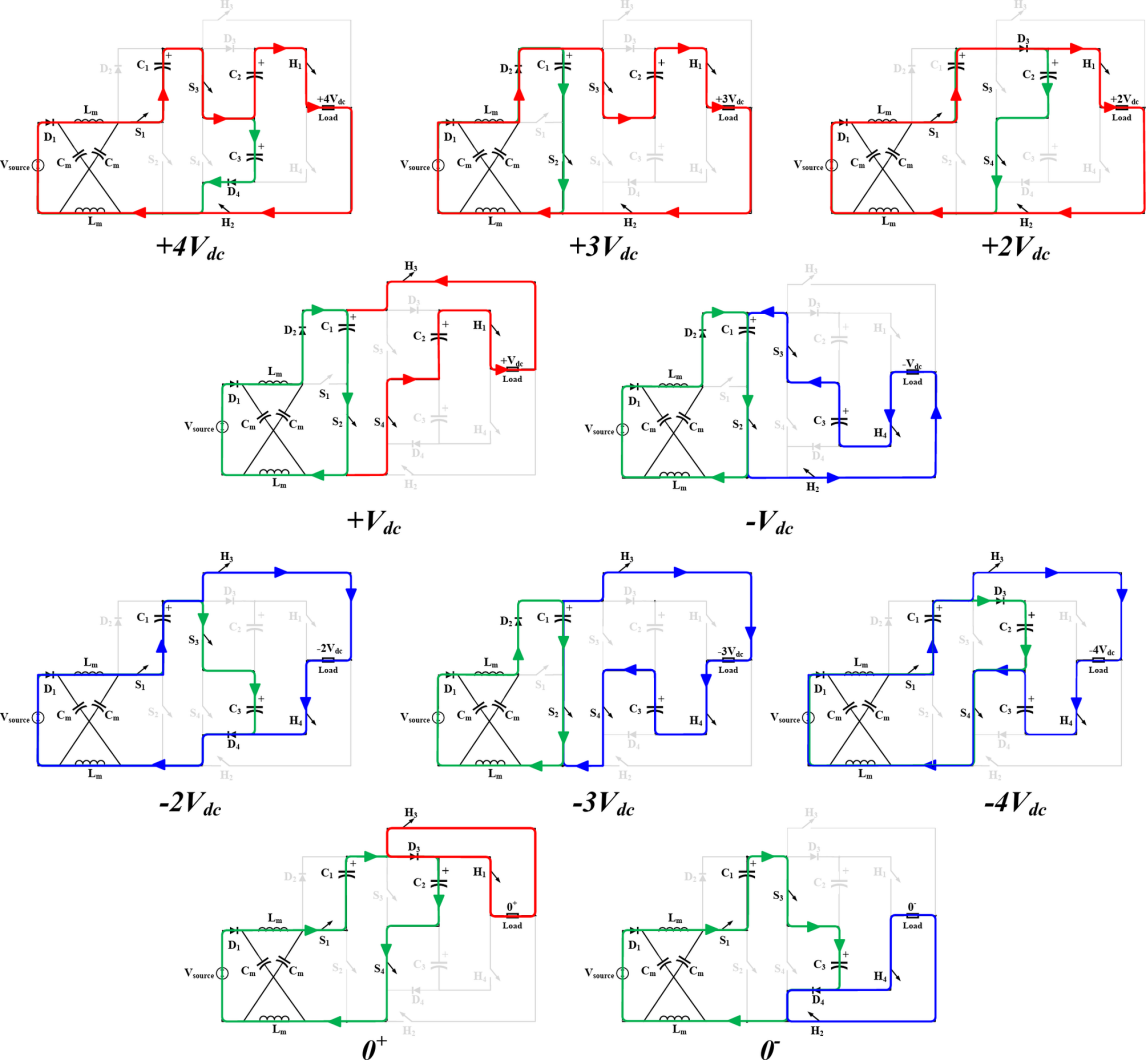
Positive half cycle modes of operation during NST states.
4. $\:{V}_{out}=+{4V}_{dc}$: $\:{S}_{1},{S}_{3}$ are switched ON to cascade the source with both $\:{C}_{1},\:{C}_{2}$ to create an output voltage level equals the sum of all their voltages $\:{V}_{out}={V}_{dc}+{V}_{C1}+{V}_{C2}$, while charging $\:{C}_{3}$ with $\:2{V}_{dc}$ from the source and $\:{C}_{1}$ through $\:{D}_{4}$.
5. $\:{V}_{out}={0}^{\pm\:}$ : the operation is divided into two modes, to charge either $\:{C}_{2}$ or $\:{C}_{3}$ with $\:2{V}_{dc}$ from the supply and $\:{C}_{1}$, while maintaining zero voltage at the output by switching ON $\:{H}_{1},{H}_{3}$ or $\:{H}_{2},{H}_{4}$.

###  Operation in ST states

**Fig. 3:**
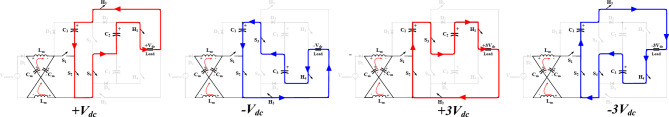
Modes of operation during ST states.

During ST states, both $\:{S}_{1},\:{S}_{2}$ are switched ON at the same time to charge the inductors within the impedance network ($\:{L}_{m1}$, $\:{L}_{m2}$). In order to evenly insert the ST states within the regular SC-MLI NST states, ST states can be inserted whenever $\:{S}_{2}$ is ON. As a result, the ST states can be realized during the output voltage levels of {$\:\pm\:{V}_{dc},\:\pm\:3{V}_{dc}\}$. Figure 3 illustrates the modes of operation during ST states. As shown, both $\:{S}_{1},{S}_{2}$ are switched ON to create a ST, while the rest of the SC network create the required output voltage level during these states. For instance, during $\:{V}_{out}=3{V}_{dc}$, $\:{S}_{3}$ is switched ON to cascade both $\:{C}_{1}$, $\:{C}_{2}$ to supply the load through $\:{H}_{1},\:{H}_{2}$ to create an output voltage level of $\:\:3{V}_{dc}$ as $\:{V}_{out}={V}_{C1}+{V}_{C2}$. During $\:{V}_{out}=-{V}_{dc}$, $\:{S}_{3}$ is switched ON to subtract $\:{\{V}_{C2},\:{V}_{C3}\}$ and reverse the output voltage polarity using $\:\{{H}_{2},\:{H}_{4}\}$. Assuming a symmetrical LC impedance network as it has the same capacitance and inductance ($\:{C}_{m1}={C}_{m2}$, and $\:{L}_{m1}={L}_{m2}$).

The dc-link voltage of the front-end ZS network can be calculated as follows:

1 $$\:{V}_{dc-link}=B{V}_{source}\:$$

Where $\:B$ refers to the boost factor, and $\:{V}_{source}$ is the supply DC voltage.

2 $$\:B=\frac{{T}_{sw}}{{T}_{1}-{T}_{0}}=\frac{1}{1-2\left({T}_{0}{/T}_{sw}\right)}=\frac{1}{1-2d}\:$$

Where $\:{T}_{sw},{T}_{1},\:{T}_{o}$, and $\:d\:$refer to the total switching period, total NST period, total ST period, and ST duty ratio, respectively. The impedance network inductors current ripple $\:{\Delta\:}{i}_{Lm}$, and the capacitors voltage ripple $\:{\Delta\:}{V}_{Cm}$ can be given as follows:

3 $$\:{\Delta\:}{i}_{Lm}=\frac{d{T}_{sw}{V}_{c}}{L},\:\:\:\:\:\:\:\:\:\:\:\:\:\:{\Delta\:}{V}_{Cm}=\frac{d{T}_{Sw}{I}_{L}}{C}\:$$

##  Modulation strategy and boosting calculations

### Modulation strategy

**Fig. 4:**
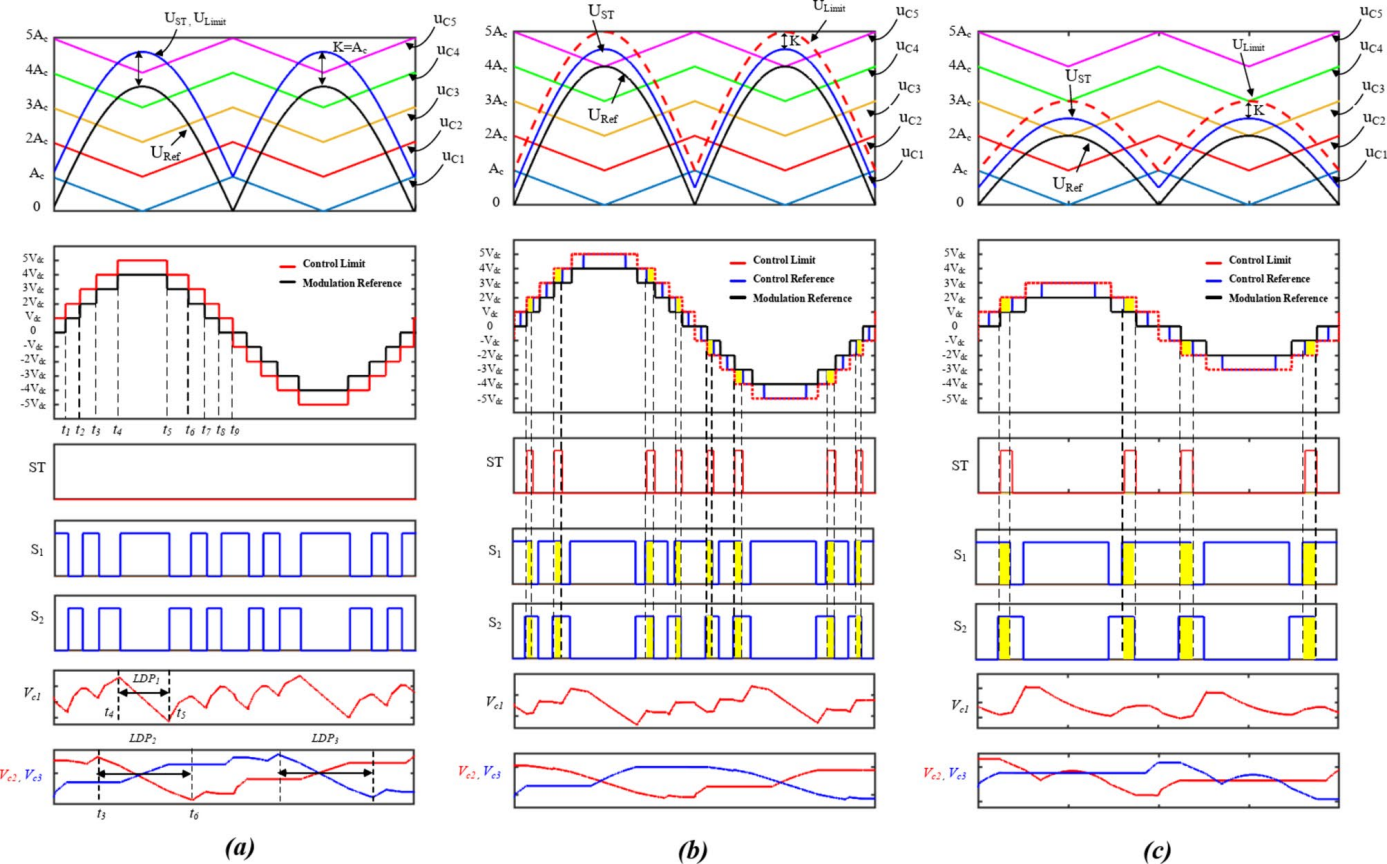
Modulation scheme of the proposed topology at: (a) $\:{M}_{a}=0.9,\:{R}_{ST}=0$, (b) $\:{M}_{a}=1,\:{R}_{ST}=50\%$, and (c) $\:{M}_{a}=0.5,\:{R}_{ST}=50\%$

In order to fulfill the required switching pattern previously described in Table 1, LS-SPWM modulation scheme is adopted. However, inserting ST states within specific voltage levels requires further considerations. As illustrated in Fig. 4, four triangular carrier signals $\:\{{u}_{c1},{u}_{c2},{u}_{c3},{u}_{c4}\}$ with equal amplitude of $\:{A}_{c}$ are level shifted to cover the amplitude of a reference sinusoidal signal $\:{U}_{ref}$. The continuous comparison between the reference signal and the carrier signals results in nine distinct control signals which refer to the output nine voltage levels. However, to evenly insert the ST states while applying LS-SPWM in the required voltage levels {$\:\pm\:{V}_{dc},\:\pm\:3{V}_{dc}\}$, a modified modulation scheme is developed.

As the total time duration of the ST in voltage levels {$\:\pm\:{V}_{dc},\:\pm\:3{V}_{dc}\}$ is not constant and varies depending on the utilized modulation index $\:{M}_{a}$. The modified ST control strategy depends on controlling the ST/NST time ratio $\:{R}_{ST}$ during each specific voltage level. This approach is implemented utilizing a sinusoidal ST control signal $\:{U}_{ST}$ that is shifted from the reference modulation signal $\:{U}_{ref}$, and a fifth carrier signal $\:{u}_{5}$. The comparison between $\:{U}_{ST}$ and all the carrier signals generates another control reference for a 11-level voltages. Changing the shifting value of $\:{U}_{ST}$ from $\:{U}_{ref}$ controls the overlap between the modulation reference and control reference. The ST control signals are generated whenever there is an overlap in the voltage levels during which a ST is implemented. For the present case a ST is required whenever there is an overlap between the two references in the following voltage levels {$\:\pm\:{V}_{dc},\:\pm\:3{V}_{dc}\}$.

For example, Fig. 4a illustrates the modulation scheme at $\:{M}_{a}=0.9$, and $\:{R}_{ST}=0$. As indicated, shifting $\:{U}_{ST}$ by $\:{A}_{c}$ results in no overlap between the voltage levels, and hence no ST is applied. Reducing the shifting of $\:{U}_{ST}$ creates an overlap between the voltage levels of the two reference signals, and hence a ST is applied. Figure 4b illustrates the case with $\:{M}_{a}=1$, and $\:{R}_{ST}=50\%$, indicating that the ST/NST time ratio during {$\:\pm\:{V}_{dc},\:\pm\:3{V}_{dc}\}$ is $\:50\%$, approximately.

Further reduction of $\:{U}_{ST}$ result in higher ST/NST time ratio until its amplitude is equal to $\:{U}_{ref}$. Operating in such condition results in a ST during the whole ST voltage levels.

Figure 4c, illustrates applying $\:{R}_{ST}=50\%$ during low modulation index of $\:{M}_{a}=0.5$, indicating that ST can still be inserted at low $\:{M}_{a}$ at {$\:\pm\:{V}_{dc}\}$, as the higher voltage levels are removed.

The ST time ratio during {$\:\pm\:{V}_{dc},\:\pm\:3{V}_{dc}\}$ can be calculated as follows:

4 $$\:{R}_{st}=\frac{k}{{A}_{c\:}}=1-\left(\frac{{U}_{ST}-{U}_{ref}}{{A}_{c}}\right)$$

Where $\:k$ denotes the sifting value of $\:{U}_{ST}$ from $\:{U}_{ref}$.

**Fig. 5:**
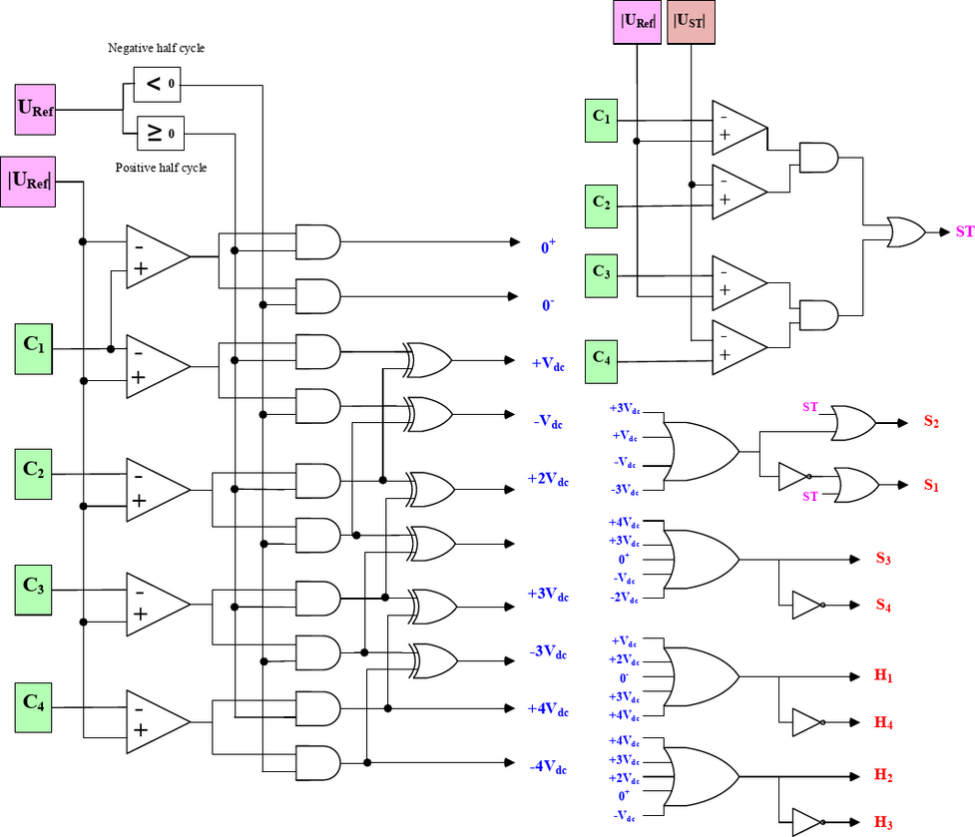
Modulation logic circuit.

Figure 5 shows the digital logic design required to apply the aforementioned modulation strategy. It can be observed that the ST signal is generated during the overlap between the modulation reference and control reference which can be represented as follows:

5 $$\:ST\:Conditions\left\{\begin{array}{c}{U}_{ref}\ge\:{u}_{c1}\:\:\:\:AND\:\:{\:\:\:U}_{ST}\le\:{u}_{C2}\\\:{U}_{ref}\ge\:{u}_{c3}\:\:\:\:AND\:\:\:\:\:{U}_{ST}\le\:{u}_{c4}\end{array}\right.$$

###  Boosting calculations

As the topology utilizes an impedance network with a ST capability, the dc-link voltage $\:{V}_{dc-link}$ supplied to the SC-MLI can be calculated based on $\:\left(2\right)$. However, to calculate the overall ST duty ratio ($\:d$) as seen from the source and ZS-network prospective. ST time ratio with respect to whole cycle time of the fundamental modulating signal $\:{U}_{ref}$ needs to be taken into consideration. Consequently, $\:d$ can be expressed as follows:

6 $$\:d=\left(\frac{{\Delta\:}{T}_{\pm\:1}+{\Delta\:}{T}_{\pm\:3}}{0.5{T}_{m}}\right){R}_{st}=\left(\frac{{\Delta\:}{T}_{1}+{\Delta\:}{T}_{3}}{0.25{T}_{m}}\right){R}_{st}\:$$

Where $\:{\Delta\:}{T}_{1}$, and $\:{\Delta\:}{T}_{3}$ denote the time duration of $\:{V}_{dc}$, and $\:3{V}_{dc}$ voltage levels, respectively during a whole fundamental modulating cycle time $\:{T}_{m}$.

**Fig. 6:**
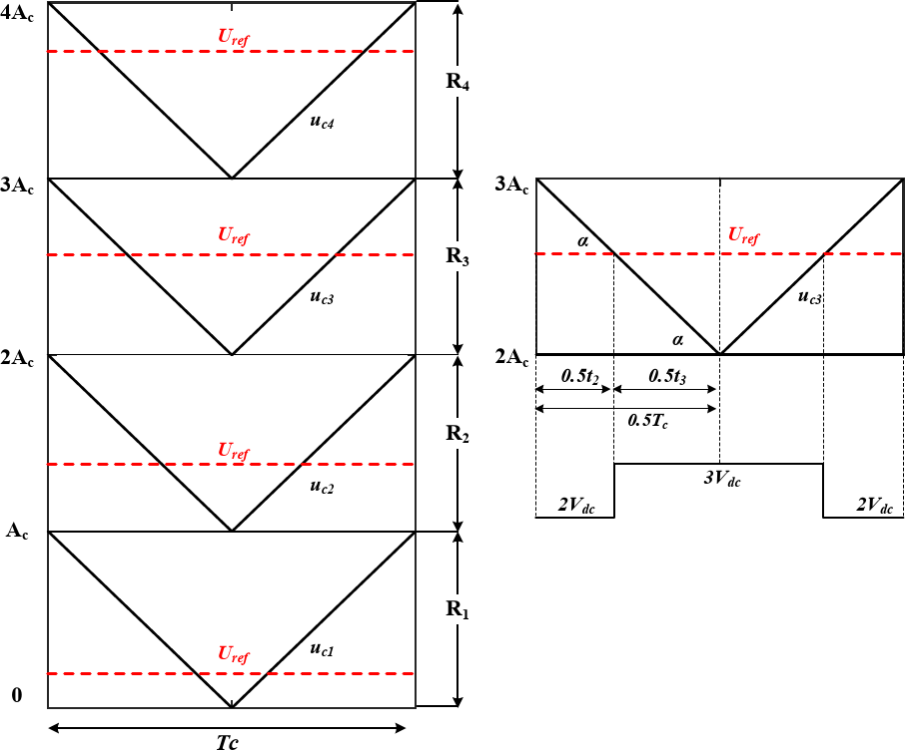
Duty ratio calculations for each voltage level.

In order to calculate $\:\{{\Delta\:}{T}_{1},\:{\Delta\:}{T}_{3}\}$, a time-based modulation function $\:\gamma\:\left(t\right)$ for each voltage in different modulation regions is defined. Figure 6 shows that the modulation can be divided into four modulation regions $\:\{{R}_{1},{R}_{2},{R}_{3},{R}_{4}\}$^28,29^. For simplicity, the mathematical analysis is applied for one carrier signal period $\:{T}_{c}$.

For example, the voltage level time ratio for $\:\{2{V}_{dc},3{V}_{dc}\}$ during the third region $\:{R}_{3}$ can be derived as follows:

7 $$\:\text{tan}\alpha\:=\:\frac{3{A}_{c}-2{A}_{c}}{0.5{T}_{c}}=\frac{3{A}_{c}-{U}_{ref}}{0.5{t}_{2}}=\frac{3{A}_{c}-4{A}_{c}{m}_{a}\text{sin}\left({\omega\:}_{m}t\right)}{0.5{t}_{2}}$$

8 $$\:{\gamma\:}_{{R}_{3},2{V}_{dc}}=\frac{{t}_{2}}{{T}_{c}}=3-4{m}_{a}\text{sin}{(\omega\:}_{m}t)$$

9 $$\:{\gamma\:}_{{R}_{3},3{V}_{dc}}=\frac{{t}_{3}}{{T}_{c}}=1-{\gamma\:}_{{R}_{2},2{V}_{dc}}=-2+4{m}_{a}\text{sin}\left({\omega\:}_{m}t\right)$$

Where $\:{\omega\:}_{m}$ is the reference signal angular frequency, while $\:{\gamma\:}_{{R}_{3},2{V}_{dc}},\:$and $\:{\gamma\:}_{{R}_{3},3{V}_{dc}}$ denote the voltage level duty ratio of $\:2{V}_{dc}$, and $\:3{V}_{dc}$ during $\:{R}_{3}$, respectively. Applying the previous mathematical analysis for all the voltage levels during all modulation regions, the time ratio for the voltage levels with respect to their modulation regions can be summarized as follows:

10 $$\:{\gamma\:}_{{R}_{1},0}\:\left(t\right)=1-4{m}_{a}\text{sin}\left({\omega\:}_{m}t\right)$$

11 $$\:{\gamma\:}_{{{R}_{i},V}_{dc}}\left(t\right)=\left\{\begin{array}{c}4{m}_{a}\text{sin}{(\omega\:}_{m}t),\:\:i=1\\\:2-4{m}_{a}\text{sin}{(\omega\:}_{m}t),\:\:i=2\end{array}\right.$$

12 $$\:{\gamma\:}_{{{R}_{i},2V}_{dc}}\left(t\right)=\left\{\begin{array}{c}-1+4{m}_{a}\text{sin}{(\omega\:}_{m}t),\:\:i=2\\\:3-4{m}_{a}\text{sin}{(\omega\:}_{m}t),\:\:i=3\end{array}\right.$$

13 $$\:{\gamma\:}_{{{R}_{i},3V}_{dc}}\left(t\right)=\left\{\begin{array}{c}-2+4{m}_{a}\text{sin}{(\omega\:}_{m}t),\:\:i=3\\\:4-4{m}_{a}\text{sin}{(\omega\:}_{m}t),\:\:i=4\end{array}\right.$$

14 $$\:{\gamma\:}_{{R}_{4},4{V}_{dc}}\left(t\right)=-3+4{m}_{a}\text{sin}\left({\omega\:}_{m}t\right)$$

Based on the previous analysis $\:{\Delta\:}{T}_{1}$, and $\:{\Delta\:}{T}_{3}$ can be calculated as follows:

15 $$\:{\Delta\:}{T}_{1}=\underset{0}{\overset{{t}_{1}}{\int\:}}{\gamma\:}_{{R}_{1},{\:V}_{dc}}\:dt+\:\underset{{t}_{1}}{\overset{{t}_{2}}{\int\:}}{\gamma\:}_{{R}_{2},{\:V}_{dc}}\:dt$$

16 $$\:{\Delta\:}{T}_{3}=\underset{{t}_{2}}{\overset{{t}_{3}}{\int\:}}{\gamma\:}_{{R}_{3},{\:3V}_{dc}}\:dt+\:\underset{{t}_{3}}{\overset{{t}_{4}}{\int\:}}{\gamma\:}_{{R}_{4},{\:3V}_{dc}}\:dt$$

The integration limits can be expressed as follows:

$$\:{t}_{1}=\frac{1}{{\omega\:}_{m}}\text{asin}\left(\frac{1}{4{m}_{a}}\right),\:\:\:\:\:\:\:\:\:\:\:\:\:\:\:\:{t}_{2}=\frac{1}{{\omega\:}_{m}}\text{asin}\left(\frac{1}{2{m}_{a}}\right)$$

17 $$\:{t}_{3}=\frac{1}{{\omega\:}_{m}}\text{asin}\left(\frac{3}{4{m}_{a}}\right),\:\:\:\:\:\:\:\:\:\:\:\:\:\:\:\:{t}_{4}=\frac{0.25}{{f}_{m}}\:\:$$

From equations (10) - (17), $\:{\Delta\:}{T}_{1},\:\text{a}\text{n}\text{d}\:{\Delta\:}{T}_{3}$ can be expressed as follows:

18 $$\:{\Delta\:}{T}_{1}=\frac{4{m}_{a}}{{\omega\:}_{m}}\left\{1-2\text{cos}\left({\omega\:}_{m}{t}_{1}\right)+\text{cos}\left({\omega\:}_{m}{t}_{2}\right)\right\}+2\left({t}_{2}-{t}_{1}\right)$$

**Fig. 7:**
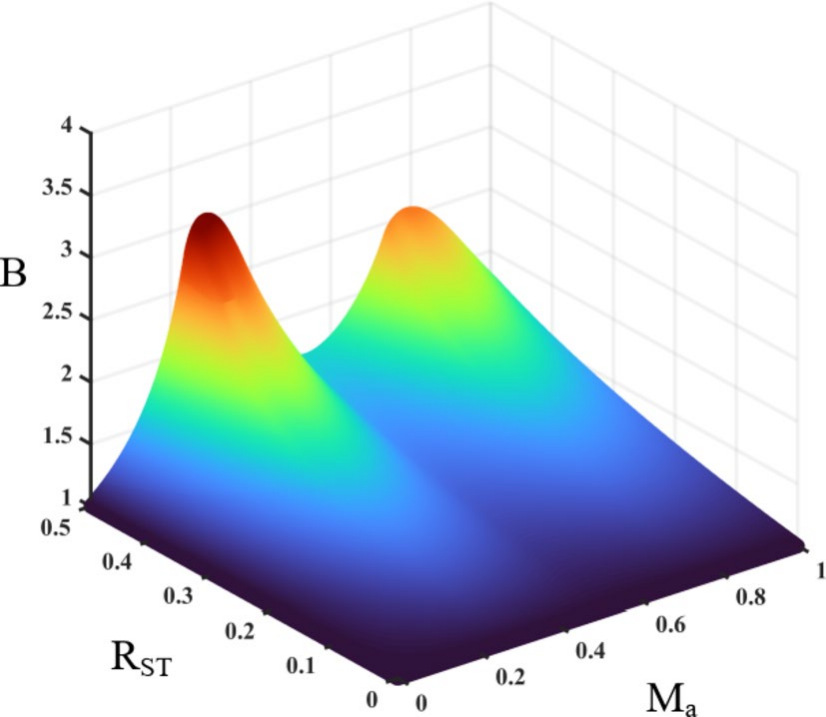
DC-link voltage gain versus the applied ST time ratio $\:{R}_{ST}$, and modulation index $\:{M}_{a}$.

19 $$\:{\Delta\:}{T}_{3}=\frac{4{m}_{a}}{{\omega\:}_{m}}\left\{\text{cos}\left({\omega\:}_{m}{t}_{2}\right)-2\text{cos}\left({\omega\:}_{m}{t}_{3}\right)\right\}+2\left({t}_{2}-3{t}_{3}+{T}_{m}\right)\:$$

This analysis and its output duty ratio formula is valid for all modulation index values $\:{m}_{a}$. However, if $\:{m}_{a}$ is reduced in a way that results in lower number of output voltage levels (i.e., 7-levels, 5-levels, … etc.), this implies that one or more of the modulation regions $\:\{{R}_{1},{R}_{2},{R}_{3},{R}_{4}\}$ are not active anymore and should be considered for the integration in equation $\:\left(17\right)$. Consequently, the ST duty ratio $\:d$ for different values of modulation index $\:{m}_{a}$ are listed in Table 2. The effect of modulation index $\:{M}_{a}$, as well as the applied ST/NST time ratio $\:{R}_{ST}$ is illustrated Fig. 7. It can be observed that the overall gain increases near modulation index $\:{M}_{a}$ of 0.8, and 0.2 due to the longer time ratio of the ST voltage levels $\:\pm\:{V}_{dc}$, and $\:\pm\:{3V}_{dc}$ which increases the overall ST duty ratio $\:d$ for the whole fundamental cycle.

## Circuit design

### Impedance network design

The design procedure for the impedance network is relative to the maximum applicable ST duty ratio for the circuit. As the inductors are charged during ST states and discharged during NST states, the inductors current ripple $\:{\Delta\:}{I}_{m}$ can be expressed as follows:

20 $$\:{\Delta\:}{I}_{Lm}=\frac{{d}_{max}{V}_{C}}{{f}_{C}L}=\frac{{d}_{max}\:\left(1-{d}_{max}\right){V}_{dc}}{\left(1-2{d}_{max}\right)L{f}_{C}}\:$$

Consequently, the minimum required inductance can be calculated as follows:

21 $$\:{L}_{m}\ge\:\left[\frac{{d}_{max}\left(1-{d}_{max}\right)}{2\left(1-2{d}_{max}\right)}\right]\:\frac{{V}_{dc}}{{f}_{c}\:{\Delta\:}{I}_{LZ,\:max}}\:\:\:$$

Where $\:{\Delta\:}{I}_{Lm,\:max\:}$ is the maximum allowable inductor current ripple, $\:{f}_{C}$ is the carrier signal frequency, and $\:{d}_{max}$ is the maximum applied ST duty ratio.

**Table 2:** Overall ST duty ratio calculation based on the modulation index.

$\:{m}_{a}$	$\:d$
$\:{m}_{a}\ge\:0.75$	$\:{R}_{st}\left\{\frac{8{m}_{a}}{\pi\:}\left[1-2\text{cos}\left({\omega\:}_{m}{t}_{1}\right)+2\text{cos}\left({\omega\:}_{m}{t}_{2}\right)-2\text{cos}\left({\omega\:}_{m}{t}_{3}\right)\right]+8{f}_{m}(-{t}_{1}+2{t}_{2}-3{t}_{3}+{0.5T}_{m})\right\}$
$\:0.5{\le\:m}_{a}\le\:0.75$	$\:{R}_{st}\left\{\frac{8{m}_{a}}{\pi\:}\left[1-2\text{cos}{(\omega\:}_{m}{t}_{1})+2\text{cos}\left(\omega\:{t}_{2}\right)\right]+8{f}_{m}(-{t}_{1}+2{t}_{2}-0.25{T}_{m})\right\}$
$\:0.25{\le\:m}_{a}\le\:0.5$	$\:{R}_{st}\left\{\frac{8{m}_{a}}{\pi\:}\left[1-2\text{cos}{(\omega\:}_{m}{t}_{1})\right]+4{f}_{m}\left(-{t}_{1}+0.5{T}_{m}\right)\right\}$
$\:{m}_{a}\le\:0.25$	$\:{R}_{st}\left\{\frac{8{m}_{a}}{\pi\:}\right\}$

The average steady state inductors current can be calculated as follows^27^:

22 $$\:\:\:\:\:\:\:\:\:\:\:\:\:\:\:\:{I}_{Lm}=\left[\frac{\left(1-{d}_{max}\right)}{\left(1-2{d}_{max}\right)}\right]\frac{{P}_{o}}{{V}_{dc}}\:$$

Where $\:{P}_{o}$ is the output power, $\:{V}_{dc}$ is the input voltage. For the Impedance network capacitors $\:{C}_{m1},\:{C}_{m2}$, assuming symmetric design (i.e., both have equal capacitance). The capacitor voltage stress $\:{V}_{Cm}$ can be calculated as follows:

23 $$\:\:\:\:\:\:\:\:\:\:\:\:\:\:\:\:\:\:\:{V}_{Cm}=\left[\frac{\left(1-{d}_{max}\right)}{\left(1-2{d}_{max}\right)}\right]{V}_{dc}\:\:$$

The capacitors voltage ripple $\:{{\Delta\:}V}_{Cm}$ can be expressed as follows:

24 $$\:{\Delta\:}{V}_{cz}=\left[\frac{{d}_{max}\left(1-{d}_{max}\right)}{2\left(1-2{d}_{max}\right)}\right]\frac{{P}_{o}}{{V}_{dc}{f}_{c}{C}_{m}}\:\:\:$$

Consequently, the minimum required capacitance can be calculated as follows:

25 $$\:{C}_{m,\:min}\ge\:\left[\frac{{d}_{max}\left(1-{d}_{max}\right)}{2\left(1-2{d}_{max}\right)}\right]\frac{{P}_{o}}{{V}_{dc}{f}_{c}{\Delta\:}{V}_{cm,\:\:max}}\:\:\:\:$$

Where $\:{\Delta\:}{V}_{cm,\:max}$, is the maximum allowable capacitor voltage ripple.

**Fig. 8:**
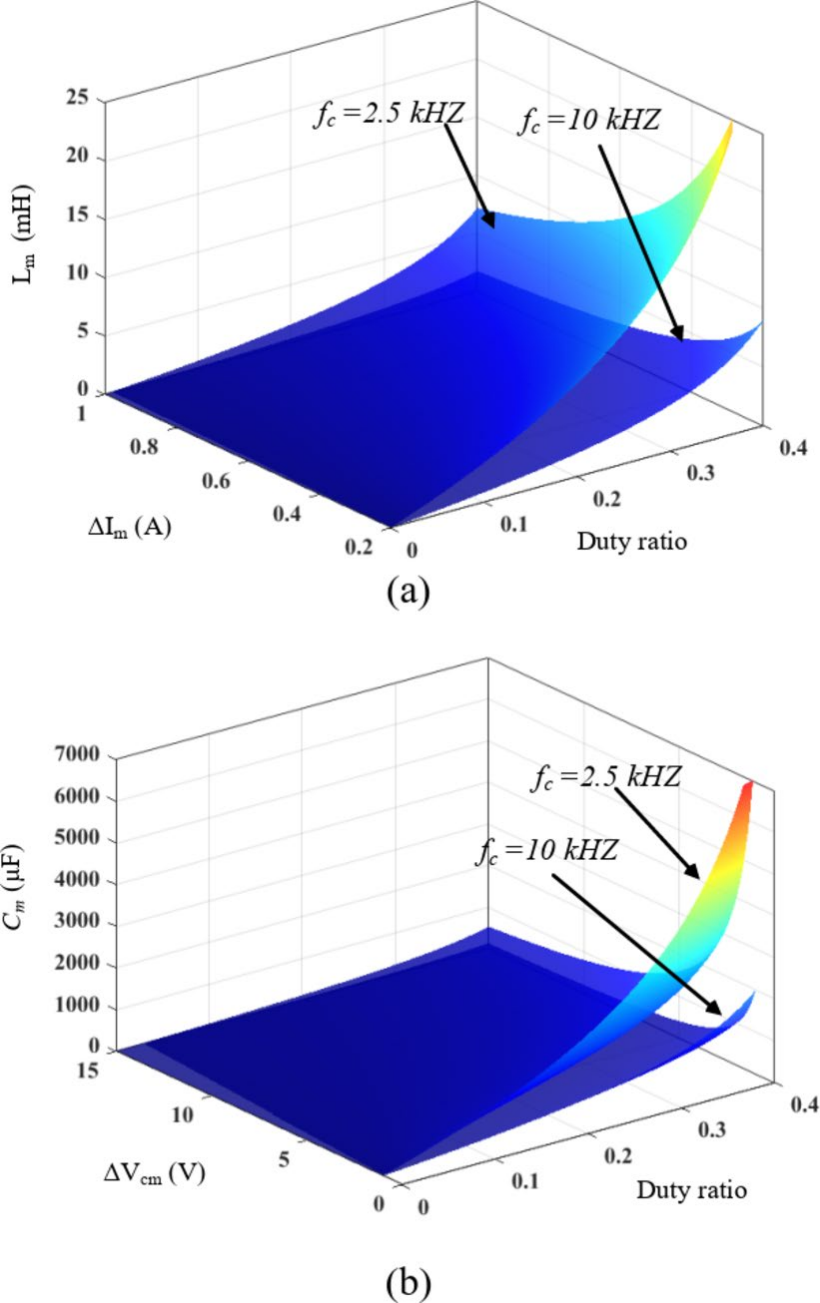
Minimum requirements for LC network versus different switching frequencies, and ST duty ratio for: (**a**) Inductors $\:{L}_{m}$, and (**b**) Capacitors $\:{C}_{m}$

Figure 8 illustrates the design requirements for the front end impedance network based on equations (25), (21). It can be observed from Fig. 8a that higher $\:{L}_{m}$ is required as the maximum applied ST duty ratio increases to keep the inductor current ripple within the permissible range. Similarly, Fig. 8b indicates higher $\:{C}_{m}$ is required to keep the capacitors voltage ripple within permissible range as the maximum ST duty ratio increases. It is worth noting that the switching frequency (i.e. carrier frequency $\:{f}_{c}$) contributes effectively to the design requirements. Both $\:{L}_{m}$ and $\:{C}_{m}$ can be greatly reduced with higher switching frequencies. However, this comes at the cost of higher switching losses, indicating a tradeoff between the network size and the inverter efficiency as well as its switching capabilities.

### Switched capacitors design

To ensure a proper operation for the SC units, and to avoid undercharging.

The SC are designed based on the longest discharge period (LDP), the peak load current, and the load power factor^30^. Considering operating in NST, and based on the voltage profiles of $\:{C}_{1},\:{C}_{2}$, and $\:{C}_{3}$ indicated in Table 1, the maximum charge required by the capacitors during resistive load can be calculated as follows:

26 $$\:{{\Delta\:}Q}_{C1,Max}={\int\:}_{{t}_{4}}^{{t}_{5}}\text{‍}{I}_{Load}dt=\frac{4{V}_{dc}}{{R}_{L}}\left[{t}_{5}-{t}_{4}\right]$$

27 $$\:{{\Delta\:}Q}_{C2,3Max}={\int\:}_{{t}_{3}}^{{t}_{6}}\text{‍}{I}_{Load}dt=\frac{2{V}_{dc}}{{R}_{L}}\left[3\left({t}_{4}-{t}_{3}\right)+2\left({t}_{5}-{t}_{4}\right)\right]$$

Whereas for inductive loads, the maximum capacitors charge can be expressed as follows:

28 $$\:{{\Delta\:}Q}_{C1,Max}={\int\:}_{{t}_{4}}^{{t}_{5}}\text{‍}{I}_{{\:l}_{Max}}sin\left(2\pi\:{f}_{m}t-\varphi\:\right)dt=\:\:\frac{{I}_{l,Max}}{2\pi\:{f}_{m}}\left[\text{c}\text{o}\text{s}(2\pi\:{f}_{m}{t}_{4}-\varphi\:)-\text{c}\text{o}\text{s}(2\pi\:{f}_{m}{t}_{5}-\varphi\:)\right]$$

29 $$\:{{\Delta\:}Q}_{C2,3Max}={\int\:}_{{t}_{3}}^{{t}_{6}}\text{‍}{I}_{{\:l}_{Max}}sin\left(2\pi\:{f}_{m}t-\varphi\:\right)dt=\:\:\:\frac{{I}_{l,Max}}{2\pi\:{f}_{m}}\left[\text{c}\text{o}\text{s}(2\pi\:{f}_{m}{t}_{3}-\varphi\:)-\text{c}\text{o}\text{s}(2\pi\:{f}_{m}{t}_{6}-\varphi\:)\right]$$

Where, $\:{I}_{{l}_{Max}},\:\varphi\:$, and $\:{f}_{m}$ denote the maximum load current, the phase shift between the load voltage and current, and the reference signal frequency, respectively. Considering a high carrier frequency, the time limits can be calculated as follows:

30 $$\:{t}_{4}=\frac{{\text{s}\text{i}\text{n}}^{-1}\left(\frac{3}{4{\text{m}}_{\text{a}}}\right)}{2\pi\:{f}_{m}}{t}_{5}=\left(\frac{{T}_{m}}{2}-{t}_{4}\right)$$

31 $$\:{t}_{3}=\frac{{\text{s}\text{i}\text{n}}^{-1}\left(\frac{2}{4{m}_{a}}\right)}{2\pi\:{f}_{m}}{t}_{6}=\left(\frac{{T}_{m}}{2}-{t}_{3}\right)$$

Where $\:{T}_{m}$ is the reference switching period. Based on the previous analysis the minimum required capacitance can be expressed as follows:

32 $$\:{C}_{1\:min}\ge\:\frac{{\Delta\:}{Q}_{C1}}{{\updelta\:}\:{\text{V}}_{\text{C}1}}{C}_{\text{2,3}\:min}\ge\:\frac{{\Delta\:}{Q}_{C2}}{{\updelta\:}\:{\text{V}}_{\text{C}3}}$$

Where $\:\delta\:$ represents the maximum permissible capacitor voltage ripple.

**Fig. 9:**
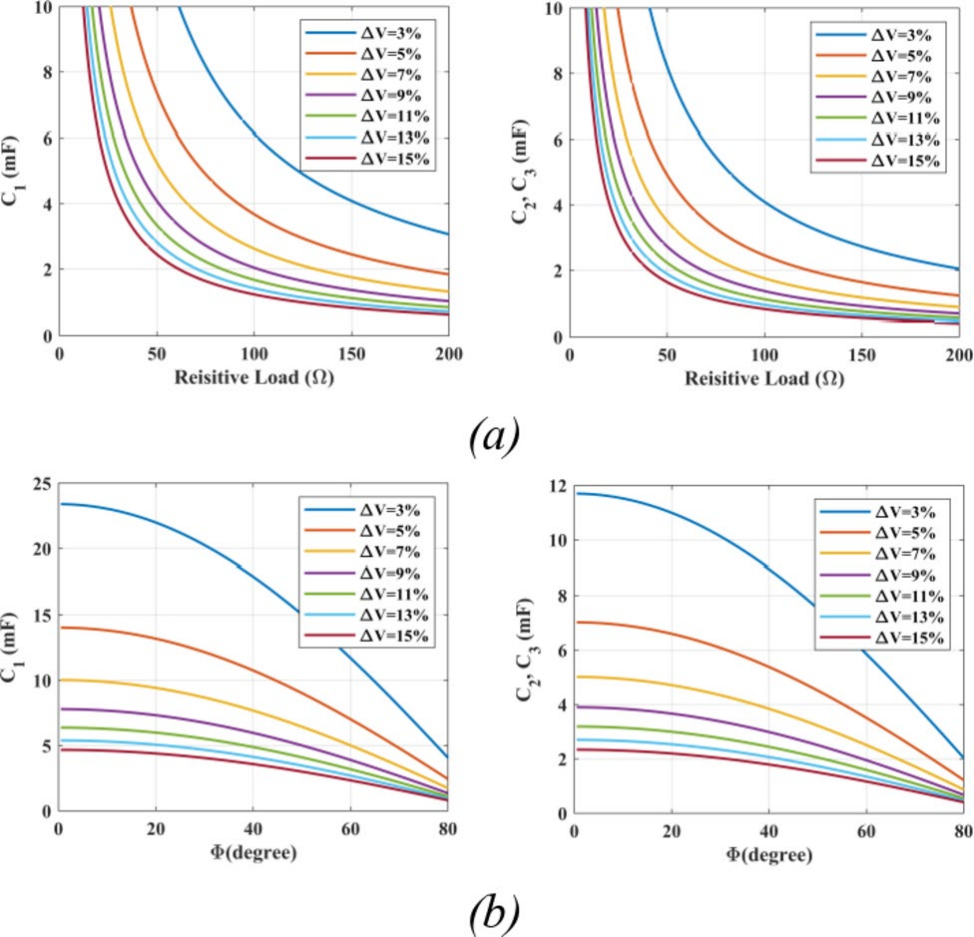
Minimum requirements for switched capacitors versus different loading conditions and permissible voltage ripples showing: (**a**) $\:{C}_{1}$, $\:{C}_{2}$, $\:{C}_{3}$ with resistive loads, and (**b**)$\:\:{C}_{1}$, $\:{C}_{2}$, $\:{C}_{3}$ with inductive load phase angles considering a maximum load current $\:{I}_{l,Max}=5\;A$.

Following equation $\:\left(32\right)$, Fig. 9 illustrates the minimum values required for the switched capacitors $\:{C}_{1},\:{C}_{2},{C}_{3}$ with respect to different loading conditions, indicating the effect of the maximum permissible voltage ripple on the capacitors size. Furthermore, the LDP effect can be clearly observed as $\:{C}_{1}$ requires higher capacitance compared to $\:{C}_{2},{C}_{3}$ considering the same load and permissible voltage ripple which is due to its longer discharge period illustrated in Fig. 4a. when it comes to inductive loads, power factor has its effect on the capacitors size which is illustrated in Fig. 9b, indicating lower capacitance requirements for larger inductive load phase angles.

**Fig. 10:**
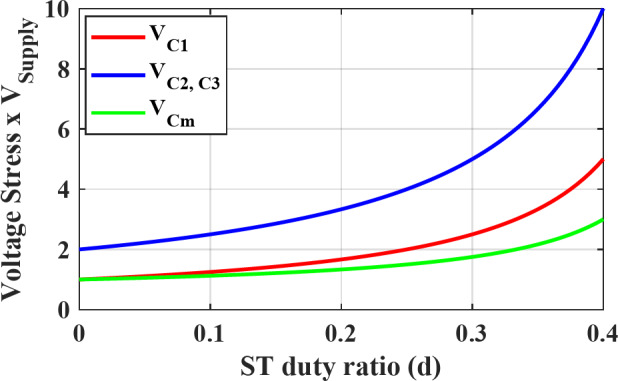
Capacitors voltage stress versus ST duty ratio.

The capacitors voltage stress per unit supply voltage can be determined from Fig. 10 against different values of ST duty ratio $\:d$. It can be noted that the LC network capacitors $\:{C}_{m}$ have the least voltage stress, while $\:{C}_{2},\:{C}_{3}$ have to withstand twice the stress of $\:{C}_{1}$.

## Power loss calculations

The main power losses present in SC-MLI are the conduction losses $\:{P}_{cond}$, the switching loss $\:{P}_{sw}$, and the capacitor ripple loss $\:{P}_{C,\:ripple}$. Conduction losses depend mainly on the load current as well as the power switches forward conduction resistances^31,32^.

On the other hand, switching losses mainly depend on the adopted modulation frequency^33^.

### Conduction losses

**Fig. 11:**
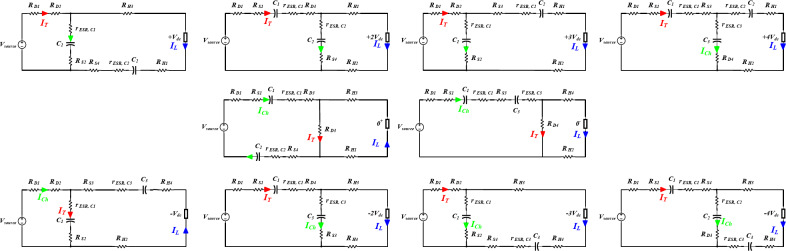
Equivalent circuit for the proposed topology in different modes of operation.

In order to represent conduction losses in different voltage levels, Fig. 11 illustrates the topology equivalent circuit for conduction power loss calculation for each voltage level including the switches forward conduction resistance $\:{R}_{s}$, the diodes forward conduction resistance $\:{R}_{D}$, and the capacitors equivalent series resistance $\:{r}_{ESR}$. The conduction losses based on Fig. 11 can be summarized as follows^34^:

33 $$\:{P}_{cond,\:+4}={I}_{T}^{2}\left({R}_{D1}+{R}_{S1}+{r}_{ESR,\:C1}+{R}_{S3}\right)+{I}_{Ch}^{2}\left({r}_{ESR,\:\:C3}+{R}_{D4}\right)+{I}_{L}^{2}({r}_{ESR,\:C2}+{R}_{H1}+{R}_{H2})$$

34 $$\:{P}_{cond,\:+3}={I}_{T}^{2}\left({R}_{D1}+{R}_{D2}\right)+{I}_{Ch}^{2}\left({r}_{ESR,\:\:C1}+{R}_{S2}\right)+{I}_{L}^{2}({R}_{S3}+{r}_{ESR,c2}+{R}_{H1}+{R}_{H2})$$

35 $$\:{P}_{cond,\:+2}={I}_{T}^{2}\left({R}_{D1}+{R}_{S1}+{r}_{ESR,\:C1}+{R}_{D3}\right)+{I}_{Ch}^{2}\left({r}_{ESR,\:\:C2}+{R}_{S4}\right)+{I}_{L}^{2}({R}_{H1}+{R}_{H2})$$

36 $$\:{P}_{cond,\:+1}={I}_{T}^{2}\left({R}_{D1}+{R}_{D2}\right)+{I}_{Ch}^{2}\left({r}_{ESR,\:\:C1}+{R}_{S2}\right)+{I}_{L}^{2}({R}_{H3}+{R}_{H1}+{r}_{ESR,C2}+{R}_{S4})$$

37 $$\:{P}_{cond,\:{0}^{+}}={I}_{Ch}^{2}\left({R}_{D1}+{R}_{S1}+{r}_{ESR,\:C1}+{R}_{D3}+{R}_{S4}+{r}_{ESR,C2}\right)+{I}_{T}^{2}\left({R}_{D3}\right)+{I}_{L}^{2}({R}_{H3}+{R}_{H1})$$

38 $$\:{P}_{cond,\:-1}={I}_{T}^{2}\left({R}_{D1}+{R}_{D2}\right)+{I}_{Ch}^{2}\left({r}_{ESR,\:\:C1}+{R}_{S2}\right)+{I}_{L}^{2}({R}_{S3}+{r}_{ESR,C3}+{R}_{H4}+{R}_{H2})$$

39 $$\:{P}_{cond,\:-2}={I}_{T}^{2}\left({R}_{D1}+{R}_{S1}+{r}_{ESR,\:C1}+{R}_{D3}\right)+{I}_{Ch}^{2}\left({r}_{ESR,\:\:C3}+{R}_{S3}\right)+{I}_{L}^{2}({R}_{H3}+{R}_{H4})$$

40 $$\:{P}_{cond,\:-3}={I}_{T}^{2}\left({R}_{D1}+{R}_{D2}\right)+{I}_{Ch}^{2}\left({r}_{ESR,\:\:C1}+{R}_{S2}\right)+{I}_{L}^{2}({R}_{H3}+{R}_{H4}+{r}_{ESR,c3}+{R}_{S4})$$

41 $$\:{P}_{cond,\:+4}={I}_{T}^{2}\left({R}_{D1}+{R}_{S1}+{r}_{ESR,\:C1}+{R}_{S3}\right)+{I}_{Ch}^{2}\left({r}_{ESR,\:\:C2}+{R}_{D3}\right)+{I}_{L}^{2}({R}_{H3}+{R}_{H4}+{r}_{ESR,C3})$$

Where $\:{I}_{L}$, $\:{I}_{Ch}$,$\:{I}_{T}$ denote the load current, the capacitor charging current, and the total current. It is worth noting that the number of conducting switches in any voltage level does not exceed four power switches resulting in lower conduction losses.

### ZS-Network losses

considering the power losses associated with LC network, the inductor power loss can be classified into conduction winding losses and core losses^35^. Due to the inductor current ripples, the inductor winding losses $\:{P}_{L,\:Winding}\:$can be further classified into DC conduction loss $\:{P}_{dc}$, and AC conduction loss $\:{P}_{ac}$, which can be calculated as follows:

42 $$\:{P}_{L,\:Winding}={P}_{ac}+{P}_{dc}$$

43 $$\:{P}_{dc}={I}_{dc}^{2}{R}_{dc}$$

44 $$\:{P}_{ac}={I}_{ac}^{2}{R}_{ac}=\frac{1}{12}{\Delta\:}{I}_{L}^{2}{R}_{ac}$$

Where $\:{\Delta\:}{I}_{L}$ denotes the inductor current ripple. As for the core loss, it can be calculated as follows^36^:

45 $$\:{P}_{Core,\:L}=G{V}_{e}$$

Where $\:G$ is the core loss coefficient and $\:{V}_{e}$ is the core volume.

### Switching losses

As the switching losses $\:{P}_{sw}$ depends on the switches turn ON time $\:{t}_{on}$, and turn OFF time $\:{t}_{off}$^31^. The switching losses can be expressed as follows:

46 $$\:{P}_{sw}=\frac{1}{6}{V}_{sw}{I}_{sw}{f}_{sw}\left({N}_{on}{t}_{on}+{N}_{off}{t}_{off}\right)\:\:\:$$

Where, $\:{N}_{on}$ and $\:{N}_{off}$ represents the number of ON and OFF switching transitions per cycle time, and $\:{V}_{sw}$ is the switch blocking voltage.

### Capacitor ripple losses

The capacitor ripple losses due to the capacitor’s voltage fluctuations can be calculated as follows^31^:

47 $$\:\:\:\:\:\:\:\:\:\:\:\:\:{P}_{C,\:ripple}=\frac{1}{2{T}_{m}}\text{C}{\left({\Delta\:}{v}_{C,\:\:ripple}\right)}^{2}$$

Where $\:{\Delta\:}{v}_{C,\:\:ripple}$, is the capacitor voltage ripple, and $\:{T}_{m}$ denote the fundamental cycle time.

###  Efficiency analysis

A $\:1000\:W$ model is simulated using Altair PSIM software to investigate the topology losses. A (CM600HA-24 A) thermal model is utilized for the power switches with body diodes, whereas (DFM100PXM33-F000) thermal model is utilized for the discrete diodes. The conduction losses due to the capacitors and inductors are considered based on their parasitic resistances^36^.

**Fig. 12:**
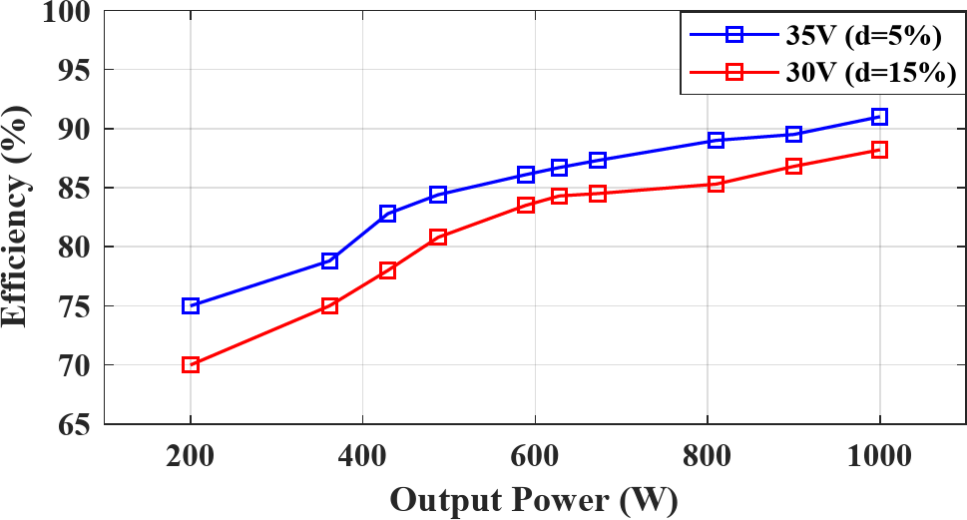
Simulated efficiency curve versus output power for different ST duty ratio.

The topology is simulated for an output power ranging from $\:200\:W$ to $\:1000\:W$ at $\:{M}_{a}=0.9$, $\:d=5\sim15\%$, $\:{V}_{in}=30\sim35\:V$, $\:{V}_{out,\:RMS}=100\:V$, $\:PF=1$, and $\:{f}_{sw}=5\:kHz$. The simulated efficiency is illustrated in Fig. 12, showing that for $\:{V}_{in}=35\:V$, and $\:d=5\%$, the efficiency is around $\:75\%\:$ at $\:200\;W$ and increasing with the output power until an efficiency around $\:91\%$ at $\:1000\:W$. Reducing $\:{V}_{in}$ requires higher ST duty $\:d$, which further increases the conduction losses through the LC network resulting in a lower efficiency around $\:88.5\%$ at $\:1000\:W$ for $\:d=15\%$.

## Comparative study

**Fig. 13:**
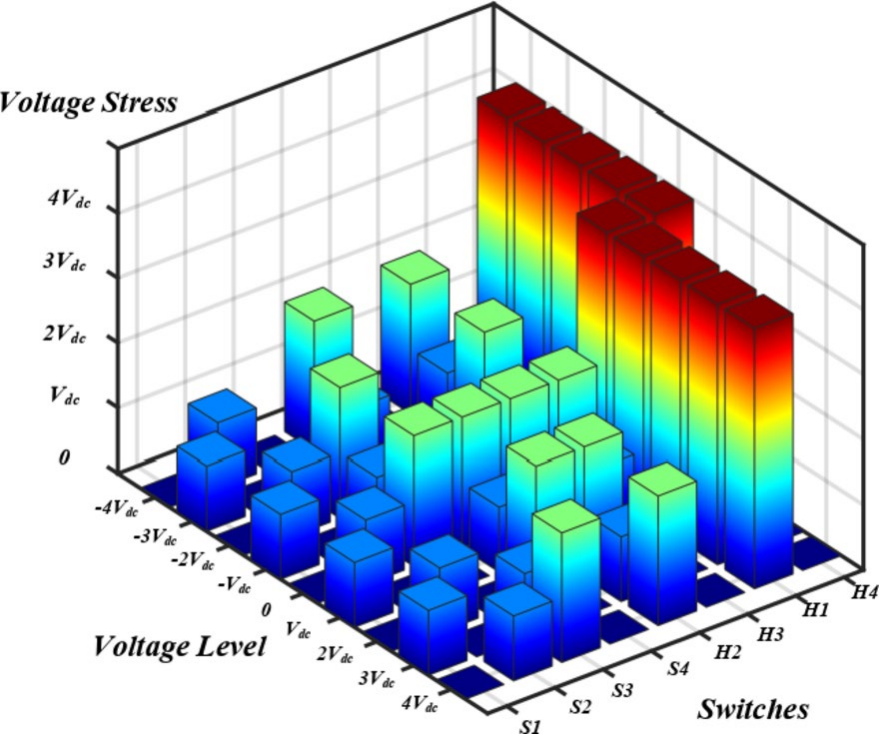
Switches blocking voltage at different voltage levels.

A comparative study is implemented to assess the proposed topology with respect to similar topologies in literature. One of the key parameters considered in the comparison is the topology Total Standing Voltage (TSV). Figure 13 illustrates switches voltage stress during different voltage levels. As illustrated, the Maximum Blocking Voltage (MBV) for both $\:{S}_{1},\:{S}_{2}$ is limited to $\:{V}_{dc-link}$. While MBV of $\:{S}_{3},{S}_{4},\:{H}_{2},\:{H}_{3}$ is limited to $\:2{V}_{dc-link}$. Only $\:{H}_{1}$, $\:{H}_{4}$ are subject to a voltage stress equals the maximum output voltage level $\:4{V}_{dc-link}$ which reduces the overall topology TSV.

The proposed topology TSV can be calculated as follows:

48 $$\:\:\:\:\:\:TSV=\sum\limits_{i=1}^{n} {V}_{sw\left(i\right)}$$

49 $$\:TS{V}_{pu}=\frac{\sum\limits_{i=1}^{n} {V}_{sw\left(i\right)}}{{V}_{o,Max}}=\frac{18{V}_{dc-link}}{4{V}_{dc-link}}=4.5$$

Where $\:{V}_{sw\left(i\right)}$ is the maximum blocking voltage across switch $\:i$, and $\:TS{V}_{pu}$ is the TSV per maximum output voltage level.

A comparative study between the proposed topology (PT) and similar ZS-based MLI is illustrated in Table 3 in terms of the hardware requirements and passive components. The topologies in^5–7^ have no issues related to the inrush currents, as the are based on QZS-CHB MLI. However, their voltage gain is only generated by the ST of the ZS-network. Furthermore^6,7^, require more power switches to generate less number of voltage levels compared to PT. ^5^ utilizes equal number of power switches to PT.

**Table 3:** Comparative study with similar ZS based topologies.

Ref	*N*_*L*_	*N*_*SW*_	*N*_*DC*_	*N*_*C*_	*N*_*D*_	*N*_*ind*_	$\:G$	$\:SC$
^7^	5	12	2	8	6	4	$\:\frac{1}{1-2d}$	-
^5^	5	8	2	3	4	2	$\:\frac{1}{1-2d}$	-
^6^	5	10	2	2	4	2	$\:\frac{1}{1-2d}$	-
^27^	7	8	1	4	3	2	$\:\frac{3}{1-2d}$	Y
[PT]	9	8	1	5	4	2	$\:\frac{4}{1-2d}$	Y

$\:{N}_{L}$: No. voltage levels, $\:{N}_{SW}$: No. of switches, $\:{N}_{C}$: No. of capacitors, $\:{N}_{D}$: No. of diodes, $\:{N}_{ind}$: No. of inductors,, $\:G$: Voltage gain, N/A: Not applicable.

However, it requires two isolated DC sources, indicating more passive components are required for the ZS-networks. a 7-level, triple voltage gain QZS-based SC-MLI is proposed in^27^ utilizing equal power switches to PT while producing less number of voltage levels and lower voltage gain.

Table 4 illustrates the comparison results between PT and other nine level SC-based MLI. A cost function is defined similar to^37^ to present a cost comparison. The cost function can be defined as follows:

50 $$\:CF=\frac{\left({N}_{sw}+{{N}_{gd}+N}_{C}+{N}_{ind}+{N}_{D}+\alpha\:TS{V}_{pu}\right){N}_{dc}}{{N}_{level}}$$

Where $\:\alpha\:$ is a weight factor for $\:TS{V}_{pu}$ to give it more priority than component counts when $\:\alpha\:>1$.

According to Table 4, topologies in^22–26,38–40^ have higher $\:CF\:$compared to PT due to the higher hardware requirements.

**Table 4:** Comparative study with similar nine level switched capacitor based topologies.

Ref	$\:{N}_{SW}$	$\:{N}_{gd}$	$\:{N}_{C}$	$\:{N}_{D}$	$\:{N}_{ind}$	$\:{N}_{DC}$	$\:G$	$\:TS{V}_{pu}$	$\:C{F}_{\alpha\:=0.5}$	$\:C{F}_{\alpha\:=1.5}$	$\:SC$
^22^	12	12	1	0	0	2	2	5	6.11	7.22	N
^38^	18	18	4	5	1	1	4	5.50	5.42	6.03	Y
^39^	19	19	3	0	0	1	4	4.75	4.82	5.35	N
^26^	8	8	0	0	0	2	1	5.5	4.17	5.39	N
^40^	12	11	4	2	2	1	3	8	3.89	4.78	Y
^23^	14	14	2	0	0	1	4	4.75	3.60	4.13	N
^24^	13	13	2	0	1	1	2	6	3.56	4.22	Y
^25^	12	9	4	2	0	1	1	5	3.28	3.82	N
[PT]	8	8	5	4	2	1	4/(1-2d)	4.50	3.25	3.75	Y
^41^	12	12	2	0	0	1	4	5.25	3.18	3.76	N
^42^	10	9	3	3	1	1	2	4.5	3.14	3.64	Y
^43^	11	10	2	1	0	1	2	5.5	2.97	3.58	N
^44^	10	9	2	2	0	1	2	4.50	2.81	3.31	N

$\:{N}_{SW}$: No. of switches, $\:{N}_{C}$: No. of capacitors, $\:{N}_{D}$: No. of diodes, $\:{N}_{ind}$: No. of inductors, $\:{N}_{DC}$: number of dc sources, $\:G$: Voltage gain, $\:CF$: cost function, and $\:SC$: soft charging.

Furthermore, both topologies proposed in^22,26^ require two DC sources and produce lower voltage gain than PT. The topologies proposed in $\:\left[22\right],\:\left[26\right],\:\left[38\right],\:\left[40\right],\:\left[41\right],\:\left[43\right]$ have higher $\:TS{V}_{pu}$ compared to PT leading to higher switches voltage rating which increases the hardware cost. The topologies in^38,39^ produce quadruple voltage gain with lower passive elements than PT. However, the power switches requirements are significantly higher than PT, implying higher costs for power switches and more complicated gate driving circuitry. In^41^ a topology is presented with suppressed capacitor currents and lower $\:CF$ than PT due to the lower count of passive components such as capacitors and inductors.

However, it produces fixed low voltage gain compared to PT which is capable of generating flexible quadruple voltage gain. The topologies proposed in^42–44^ have lower $\:CF$ than PT due to the minimized number of passive components. However, all of them still suffer from the capacitors charging inrush currents, creating higher current stress on the power supply, and reducing the capacitors lifetime. Moreover^43,44^, have lower voltage gain compared to PT. All the topologies listed in Table 4 do not offer shoot through immunity to the system unlike PT. Hence, dead time is required which has its adverse effects on the output voltage distortion. Alternatively ST mitigation circuits can be utilized which increases the overall costs.

## Simulation and experimental results

### Simulation analysis

**Fig. 14:**
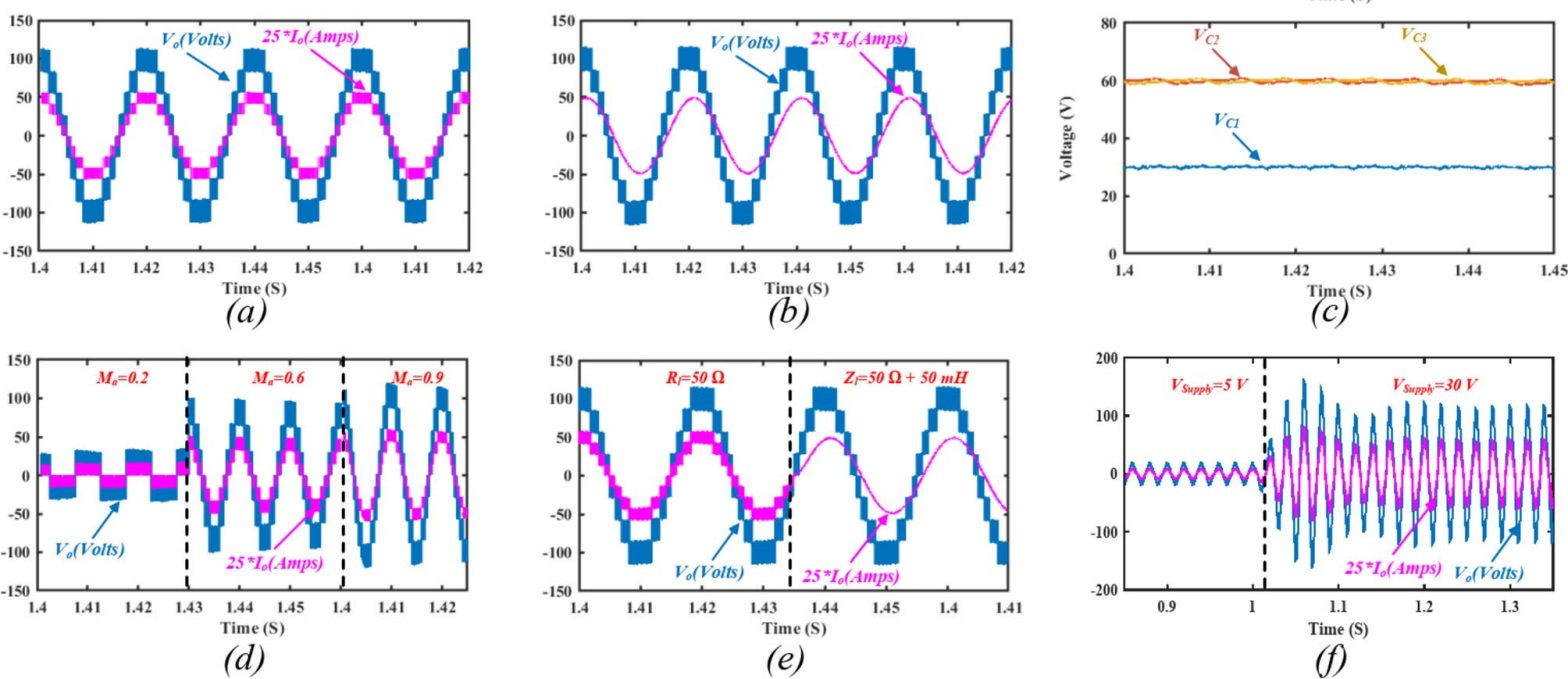
Simulation results showing: (**a**) the output voltage and load current for a resistive load of $\:{R}_{l}=50{\Omega\:}$, (**b**) the output voltage and load current for an inductive load of $\:{Z}_{l}=50{\Omega\:}+50mH$, (**c**) SC voltage profile, (**d**) a step change in $\:{M}_{a}$ from $\:0.2$, to $\:0.6$, then $\:0.9$, (**e**) a load step change from a resistive load of $\:{R}_{l}=50{\Omega\:}$, to an inductive load of $\:{Z}_{l}=50{\Omega\:}+50mH$, and (**f**) step change in the supply voltage from $\:5\;V$ to $\:30\;V$.

**Fig. 15:**
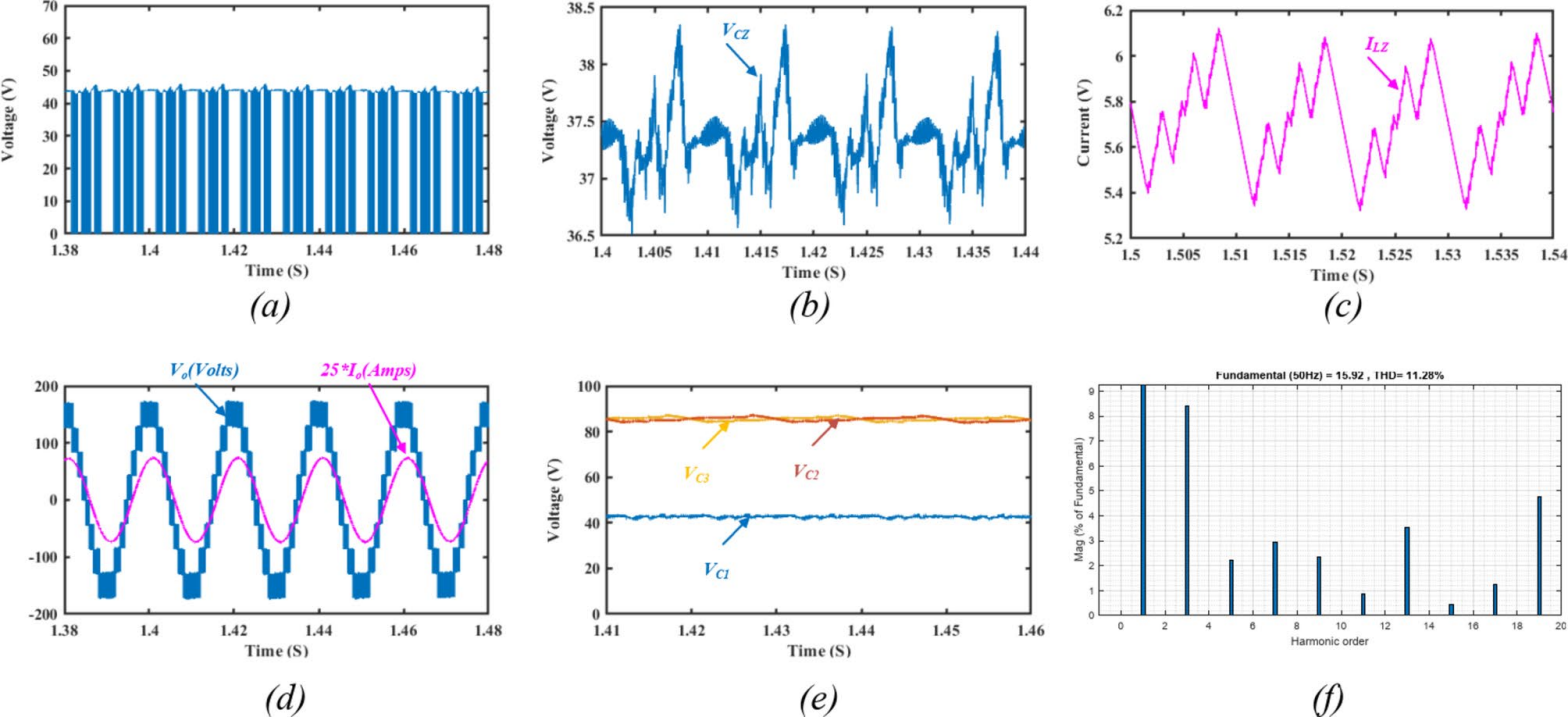
Simulation results at $\:d=15\%$ showing: (**a**) the dc-link voltage $\:{V}_{dc-link}$, (**b**) ZS capacitor voltage $\:{V}_{Cm}$, (**c**) ZS inductor current $\:{I}_{Zm}$, (**d**) the output voltage and load current for an inductive load of $\:{Z}_{l}=50{\Omega\:}+50mH$, (**e**) SC voltage profiles, and (**f**) THD for the output voltage.

The simulation study is implemented utilizing MATLAB / Simulink, a simulation model is constructed for the proposed topology. Table 5 shows the simulation model as well as the experimental prototype parameters. Figures 14 and 15 show the simulation results for the proposed topology.

**Table 5:** Simulation and experimental prototype parameters.

Parameter	Specification
$\:{V}_{dc}$	30 V
$\:{C}_{1},\:{C}_{2},\:{C}_{3}$	4200 $\:\mu\:F$, 400 V
$\:{L}_{m}$	$\:25\:mH$
$\:{C}_{m}$	1000 $\:\mu\:F$, 400 V
IGBT	CM100DY-24NF
Discrete diode	DSD-16 A 224 K
Control desk	dSPACE RTI 1104
Resistive load	$\:50{\Omega\:}$
Inductive load	$\:50mH$
Amplitude Modulation Index ($\:{M}_{a}$)	$\:0.2-1$
Carrier frequency	$\:5$kHz
Output frequency	50 Hz

First, the model is simulated for $\:d=0$, and no boosting is achieved from the Z-source network to test the SC-MLI operation. The output voltage and load current for a resistive load $\:{R}_{l}=50{\Omega\:}$ is depicted in Fig. 14a. It can be noted that the topology produces nine voltage levels with a maximum voltage level of $\:120\:V$. Hence, the topology produces quadruple voltage gain due to the aggregation of switched capacitors without applying any ST at the ZS network. Figure 14b illustrates the output voltage and load current at inductive load $\:{Z}_{l}=50{\Omega\:}+50mH$.

The switched capacitors voltage profiles are depicted in Fig. 14c, indicating that $\:{C}_{1}$ naturally balance at the dc-link voltage $\:30\:V$, while both $\:{C}_{2},\:{C}_{3}$ charge and discharge alternatively and naturally balance at twice the dc-link voltage $\:60\:V$.

In order to validate the topology stability against step changes, a step change in the amplitude modulation index $\:{M}_{a}$ is implemented in Fig. 14d.

As illustrated, the topology presents a stable response during different values of $\:{M}_{a}$. At a low modulation index $\:{M}_{a}=0.2$, the topology produces two voltage levels, while increasing $\:{M}_{a}$ to 0.6 results in 7 output voltage levels.

Figure 14e presents a load step change from resistive load of $\:{R}_{l}=50\:{\Omega\:}$ to an inductive load of $\:{Z}_{l}=50{\Omega\:}+50mH$, indicating stable output response.

A step change in the supply voltage is implemented in Fig. 14f from $\:5\:V$ to $\:30\:V$, indicating a short transient time required for the capacitors to naturally balance to their new voltage levels.

Applying ST states to the ZS-network results in boosted dc–link voltage and hence boosted output voltage. Figure 15 illustrates the simulation results for a ST duty ratio $\:d=15\%$. The dc-link voltage is illustrated in Fig. 15a, indicating a uniform distribution of the ST states during their specified voltage levels. It can be noted that the dc-link voltage reaches $\:45\;V$, fulfilling the presented calculations. Figure 15b shows the impedance network capacitor voltage profile $\:{V}_{Cm}$, indicating an average voltage of $\:37\:V$.

**Fig. 16:**
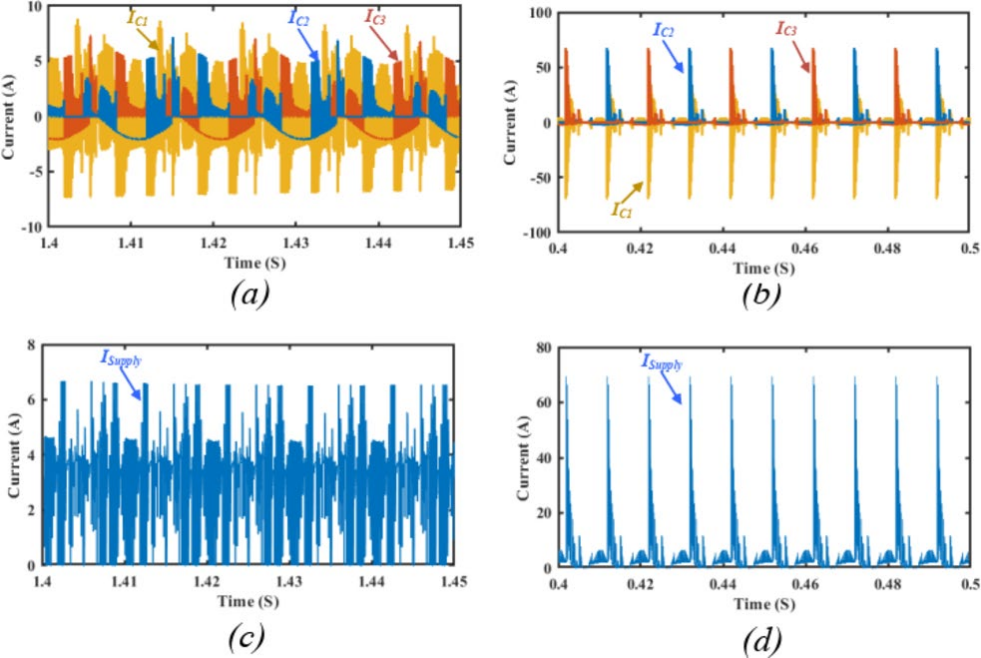
Simulation results showing: (**a**) SC currents with ZS-network, (**b**) SC currents without the ZS-network, (**c**) the supply current with ZS-network, (**d**) the supply current without the ZS-network.

The impedance network inductors current ripple $\:{I}_{Lm}$ is depicted in Fig. 15c, indicating an average inductor current of $\:5.8\:A$. The output voltage and load current are illustrated in Fig. 15d, indicating a maximum output voltage of $\:172\:V\:$approximately. Hence, the ST duty ratio resulted in an overall voltage gain of 1.45 in $\:{V}_{dc-link}$. The switched capacitors’ voltage profiles are depicted in Fig. 15e indicating that $\:{C}_{1}$ balances at $\:{V}_{dc-link}$, while both $\:{C}_{2},\:{C}_{3}$ balance at $\:2{V}_{dc-link}$. Hence, validating adequate charging and discharging states of the switched capacitors along with the ST states of the frontend impedance network. The harmonic spectrum for the output voltage is shown in Fig. 15f, indicating a THD of $\:11.28\%$.

To highlight the suppression in switched capacitors charging currents, the switched capacitors currents are illustrated in Fig. 16a. It can be observed that the charging currents are greatly suppressed compared to the condition without the ZS-network depicted in Fig. 16b. The soft charging effect on the source current is illustrated in Fig. 16c indicating the elimination of the inrush current stresses on the DC supply compared to the case without ZS-network depicted in Fig. 16d.

### Experimental results

**Fig. 17:**
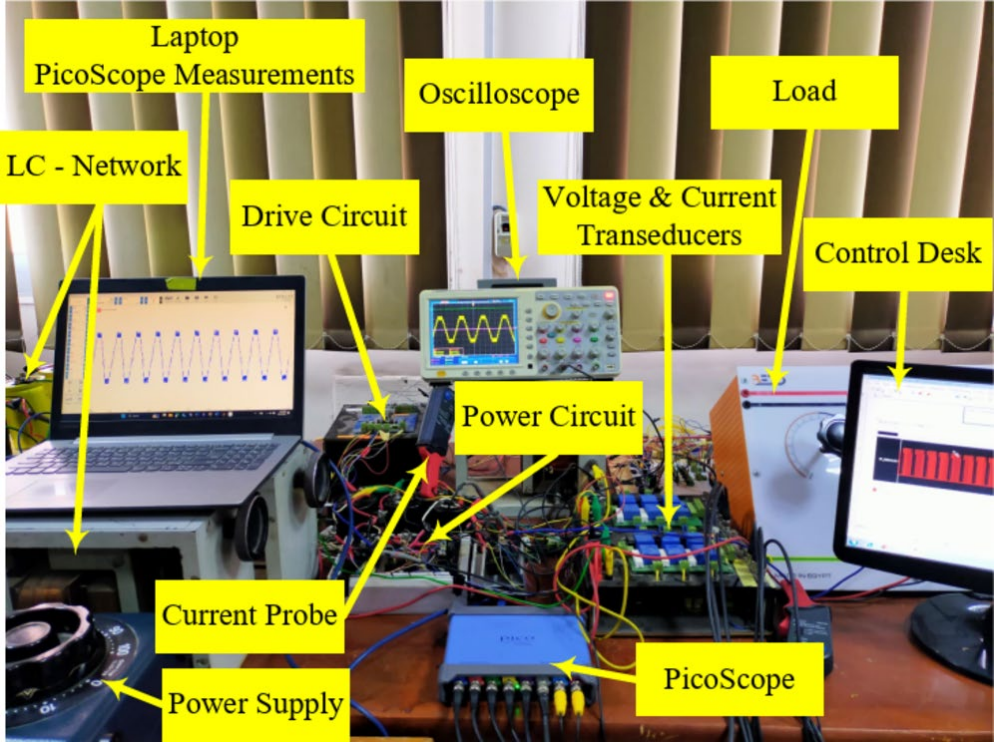
Experimental prototype.

**Fig. 18:**
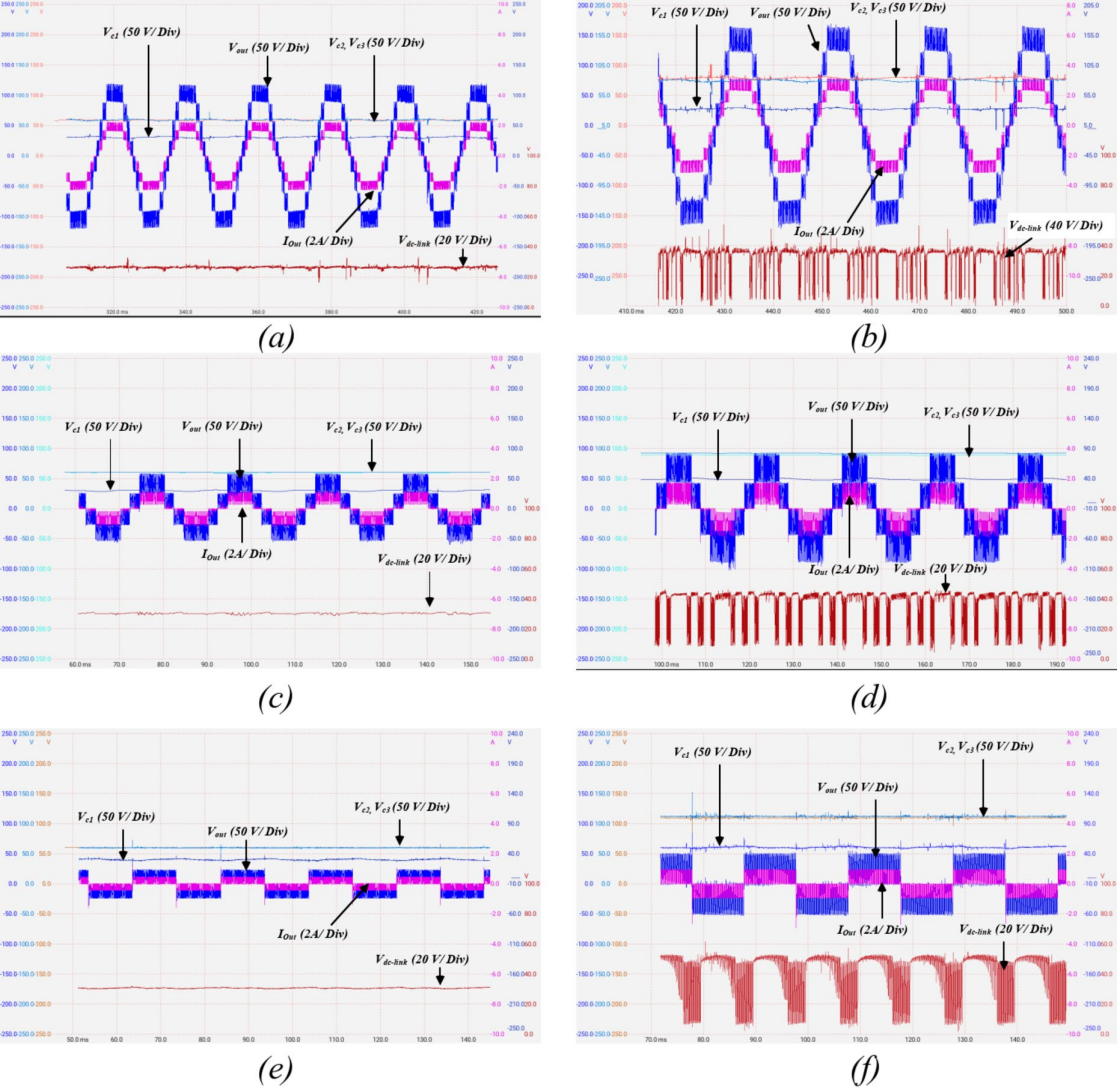
Experimental results showing output voltage and load current, switched capacitors voltage profiles, and dc – link voltage at resistive load of $\:{R}_{l}=50{\Omega\:}$ for: (**a**) $\:{M}_{a}=1$, $\:d=0$, (**b**) $\:{M}_{a}=1$,$\:d=15\%$, (**c**) $\:{M}_{a}=0.4$, $\:d=0$, (**d**) $\:{M}_{a}=0.4$, $\:d=15\%$, (**e**) $\:{M}_{a}=0.2$, $\:d=0$, and (**f**) $\:{M}_{a}=0.2$, $\:d=20\%$.

In order to validate the previous simulation results, as well as the proposed modulation strategy, an experimental prototype is built as shown in Fig. 17. The hardware utilized as well as the circuit parameters are indicated in Table 5 to match the simulation model. dSPACE 1104 is utilized to implement the required modulation scheme. TLP250 optocoupler – based gate drive is utilized for the power switches. Finally, the results are captured by Picoscope 4000 series through voltage and current transducers. To apply real-time control over the modulation index, as well as the ST duty ratio, Control Desk V4 is utilized along with dSPACE 1104. The topology is tested with different $\:{M}_{a}$ values for both NST and ST operations with its experimental results shown in Fig. 18. First the topology is tested at $\:{M}_{a}=1$ with no ST duty ratio in Fig. 18a.

The output voltage, the load current, the switched capacitors voltage profiles, and the dc-link voltage are illustrated for a resistive load of $\:{R}_{l}=50{\Omega\:}$. It can be observed that the topology generates nine-level output voltage reaching a maximum level of $\:120V$, expressing quadruple voltage gain. Furthermore, the switched capacitors balance at their required voltage levels as $\:{C}_{1}$ balances at $\:30V$, while $\:{C}_{2},{C}_{3}$ balance at $\:60V$. Figure 18b depicts the experimental results for $\:d=15\%$ at $\:{M}_{a}=1$. It can be observed that ST states are evenly distributed through the ST voltage levels $\:\{\pm\:{V}_{dc},\:\pm\:3{V}_{dc}\}$ to boost $\:{V}_{dc-link}$ by $\:40\%\:$ to reach $\:40V$. Similarly, $\:{C}_{1}$ balances at $\:{V}_{dc-link}$, while both $\:{C}_{2},\:{C}_{3}$ balance at $\:2{V}_{dc-link}$. Hence, the output voltage reaches a maximum output voltage of $\:170V$ at $\:{M}_{a}=1$, indicating better dc–link utilization. The topology and modulation scheme is tested for lower values of $\:{M}_{a}$.

Similar test is shown in Fig. 18c and d to show the topology output at $\:{M}_{a}=0.4$ without ST, and with $\:d=\:15\%$, respectively. It can be observed that despite the third voltage levels $\:\{\pm\:3{V}_{dc}\}$ have been removed, the topology is still able to boost $\:{V}_{dc-link}$ by applying ST states in $\:\{\pm\:{V}_{dc}\}$. Hence, the topology incorporated with the proposed modulation scheme is valid for the entire range of the modulation index $\:{M}_{a}$. Finally, the experimental results for low modulation index of $\:{M}_{a}=0.2$ are depicted in Fig. 18e, and f for $\:d=0$, and $\:d=20\%$, respectively. It can be observed that $\:{V}_{dc-link}$ is boosted to $\:50V$.

**Fig. 19:**
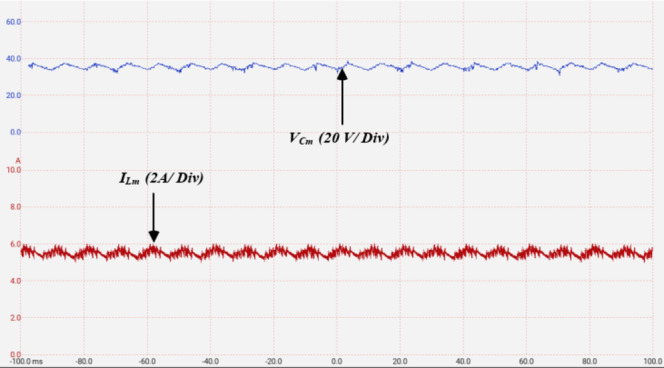
Experimental results for $\:{M}_{a}=0.9$, $\:d=15\%$ showing: The impedance network capacitor voltage, and inductor current.

Figure 19 illustrates the ZS – impedance network capacitor voltage $\:{V}_{Cm}$ and inductor current $\:{I}_{Lm}$ at $\:{M}_{a}=0.9$, and $\:d=15\%$. As indicated, $\:{V}_{Cm}$ balances at an average voltage of $\:36V$, while $\:{I}_{Lm}$ has an average value of $\:5.5\:A$.

**Fig. 20:**
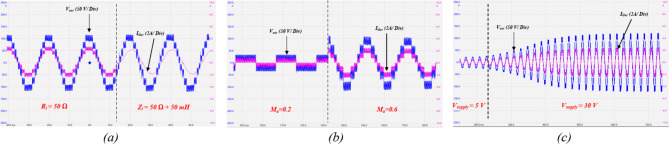
Experimental results showing the output voltage and load current during: (**a**) step change in the load from resistive load of $\:{R}_{l}=50{\Omega\:}$ to inductive load of $\:{Z}_{l}=50{\Omega\:}+50mH$, (**b**) step change in the modulation index $\:{M}_{a}$ from $\:0.2$ to $\:0.6$, and (**c**) step change in the supply voltage from $\:5\;V$ to $\:30\;V$.

The topology stability is verified experimentally through applying different step change conditions. Figure 20a shows the output voltage and load current during a load step change from resistive load of $\:{R}_{l}=50{\Omega\:}$ to inductive load of $\:{Z}_{l}=50{\Omega\:}+50mH$, indicating a stable output voltage. Figure 20b, illustrate the topology response to a step change in the modulation index $\:{M}_{a}$ from $\:0.2$ to $\:0.6$.

A step change in the supply voltage from $\:5\:V$ to $\:30\:V$ is implemented in Fig. 20c, indicating the SC naturally balance to their new voltage levels with a stable output response.

**Fig. 21:**
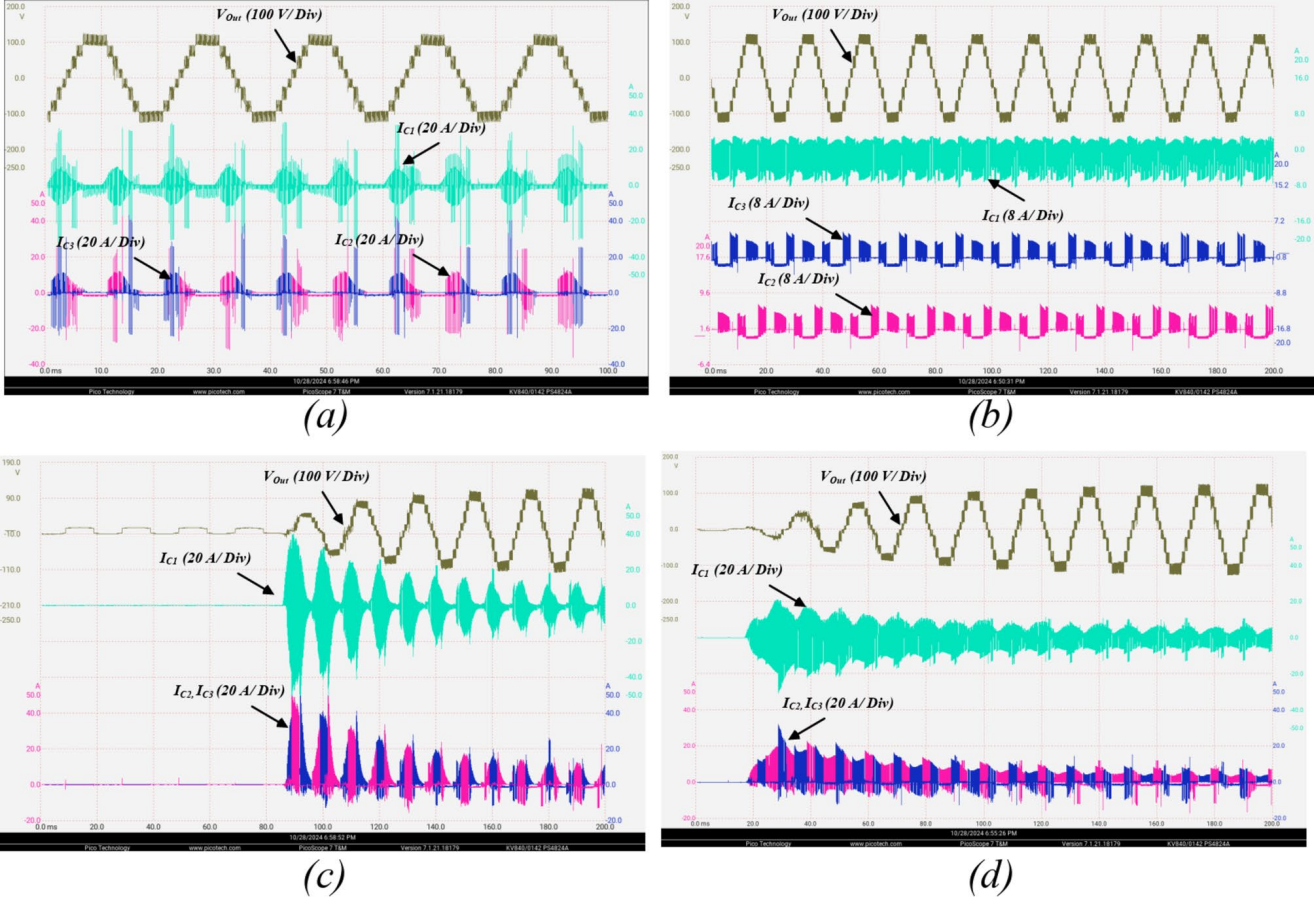
Experimental results showing the output voltage, and the switched capacitor currents for the following conditions: (**a**) capacitors charging without ZS network, (**b**) capacitors charging with ZS network, (**c**) capacitors start-up inrush without ZS network, (**d**) capacitors start-up inrush with ZS network.

The impact of ZS network can be further investigated from Fig. 21, showing its effect on the topology inrush currents. Figure 21a shows the charging currents of $\:{C}_{1},{C}_{2},\:{C}_{3}$ without the ZS network, indicating high inrush charging currents exceeding $\:18\:A$. Connecting a ZS network significantly limits the charging currents below $\:7\:A$ which is illustrated in Fig. 21b. Besides the capacitors charging current suppression, the capacitors start-up inrush currents are also investigated with and without the ZS-network. Figure 21c shows the start-up inrush current for the switched capacitors $\:{C}_{1},{C}_{2},\:{C}_{3}$ without a ZS network, indicating heavy inrush currents exceeding $\:40\:A$ at start-up, which affects the dc supply.

Figure 21d shows the soft start-up charging effect of the ZS inductors on the switched capacitors start-up inrush current. It can be observed that $\:{C}_{1}$ inrush current is significantly limited below $\:20\:A$. Similar effect can be observed for $\:{C}_{2},{C}_{3}$ with their start-up inrush currents suppressed from $\:50\:A$ to $\:25\;A$.

**Fig. 22:**
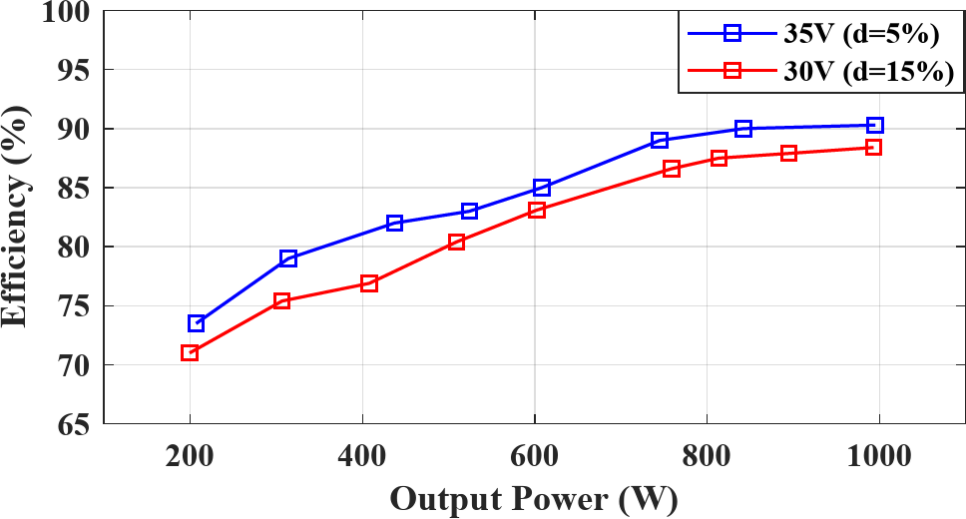
Experimental measured efficiency curve versus output power for different ST duty ratio.

Figure 22 shows the measured efficiency for the experimental setup versus the output power ranging from $\:200\:W$ to $\:1000\:W$ for different values of ST duty ratio $\:d$. For $\:{V}_{supply}=35\:V$ and $\:d=5\%$, the topology achieves $\:76\%$ efficiency at $\:200\,W$ and increasing with the output power until a steady efficiency of $\:90\%$ at $\:1000\;W$. Increasing the ST duty ratio to $\:d=15\%$ for a lower input voltage $\:{V}_{supply}=30\:V$ results in higher conduction losses in the LC inductors which reduces the overall inverter efficiency to $\:87\%$ at $\:1000\;W$.

## Conclusion

This article has presented a ZS-based SC-MLI capable of generating nine output voltage levels, with a dynamic quadruple voltage gain and suppressed capacitors inrush currents. The voltage gain can be further controlled by controlling the ZS-network ST states. A modified modulation strategy is proposed to enhance the voltage boosting capability at higher modulation index values, as well as the dc-link utilization. The topology operation is presented along with its modes of operation. A detailed mathematical analysis is presented for the boosting calculations, as well as the circuit parameters design. A simulation model has been implemented utilizing MATLAB / Simulink. The topology power losses have been investigated along with its efficiency through simulation and experimental measurements. An experimental prototype is built to verify the simulation findings. The topology has been tested for different duty ratio and modulation index values. Furthermore, the topology stability has been verified through numerous step changes in the load, the modulation index, and the output voltage. The suppression for switched capacitors inrush currents has been verified through testing the topology with and without the ZS-network.

## Data Availability

The datasets used and/or analyzed during the current study are available on request from the corresponding author.
